# Intestinal transgene delivery with native *E. coli* chassis allows persistent physiological changes

**DOI:** 10.1016/j.cell.2022.06.050

**Published:** 2022-08-04

**Authors:** Baylee J. Russell, Steven D. Brown, Nicole Siguenza, Irene Mai, Anand R. Saran, Amulya Lingaraju, Erica S. Maissy, Ana C. Dantas Machado, Antonio F.M. Pinto, Concepcion Sanchez, Leigh-Ana Rossitto, Yukiko Miyamoto, R. Alexander Richter, Samuel B. Ho, Lars Eckmann, Jeff Hasty, David J. Gonzalez, Alan Saghatelian, Rob Knight, Amir Zarrinpar

**Affiliations:** 1 Division of Gastroenterology, University of California, San Diego, La Jolla, CA 92093, USA; 2 Clayton Foundation Laboratories for Peptide Biology, The Salk Institute for Biological Studies, La Jolla, CA 92037, USA; 3 Department of Pharmacology, University of California, San Diego, La Jolla, CA 92093, USA; 4 Skaggs School of Pharmacy and Pharmaceutical Sciences, University of California, San Diego, La Jolla, CA 92093, USA; 5 VA Health Sciences San Diego, La Jolla, CA 92161, USA; 6 BioCircuits Institute, University of California, San Diego, La Jolla, CA 92093, USA; 7 Department of Bioengineering, University of California, San Diego, La Jolla, CA 92093, USA; 8 Department of Pediatrics, University of California, San Diego, La Jolla, CA 92093, USA; 9 Center for Microbiome Innovation, University of California, San Diego, La Jolla, CA 92093, USA; 10 Department of Computer Science and Engineering, University of California, San Diego, La Jolla, CA 92093, USA; 11 Lead contact

## Abstract

Live bacterial therapeutics (LBTs) could reverse diseases by engrafting in the gut and providing persistent beneficial functions in the host. However, attempts to functionally manipulate the gut microbiome of conventionally raised (CR) hosts have been unsuccessful because engineered microbial organisms (i.e., chassis) have difficulty in colonizing the hostile luminal environment. In this proof-of-concept study, we use native bacteria as chassis for transgene delivery to impact CR host physiology. Native *Escherichia coli* bacteria isolated from the stool cultures of CR mice were modified to express functional genes. The reintroduction of these strains induces perpetual engraftment in the intestine. In addition, engineered native *E. coli* can induce functional changes that affect physiology of and reverse pathology in CR hosts months after administration. Thus, using native bacteria as chassis to “knock in” specific functions allows mechanistic studies of specific microbial activities in the microbiome of CR hosts and enables LBT with curative intent.

## In brief

Native *E. coli* strains isolated from mouse stool are genetically engineered for long-term engraftment in the conventional mouse gut and enable long-term systemic effects on the host, such as improvements in insulin sensitivity in mouse models of type 2 diabetes.

## Graphical Abstract

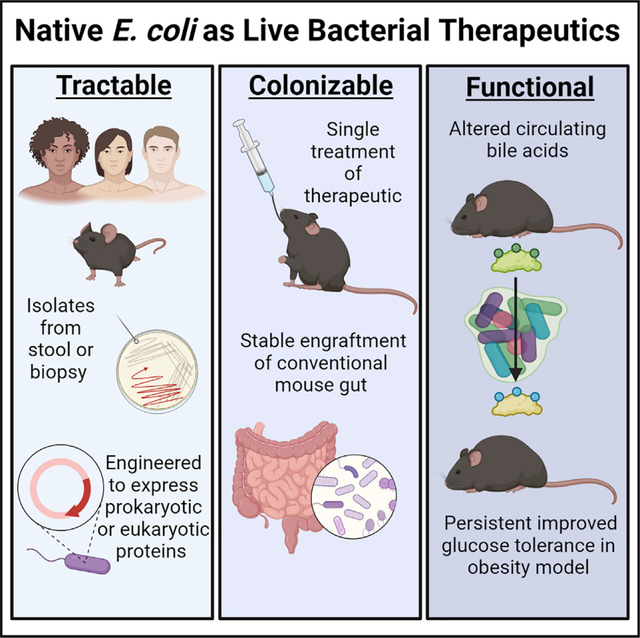

## INTRODUCTION

Gut microbes sense and condition the luminal environment over time periods measured in years. Numerous chronic human diseases, including obesity (Muscogiuri et al., 2019; Zarrinpar et al., 2014), non-alcoholic fatty liver disease (Aron-Wisnewsky et al., 2020; Saran et al., 2020), type 2 diabetes (Gurung et al., 2020; Zarrinpar et al., 2014), atherosclerosis (Allaband et al., 2021; Jie et al., 2017; Xue et al., 2021), polycystic ovary syndrome (Rizk and Thackray, 2021), inflammatory bowel disease (Kostic et al., 2014), and cancer (Sepich-Poore et al., 2021), have protracted symptomology and have consequently been targets of potential engineered, live bacterial therapeutics (LBTs). LBTs can also be designed to express functions that address monogenic inborn errors of metabolism (e.g., phenylalanine metabolism in patients with phenylketonuria) and other orphan diseases (Isabella et al., 2018; Puurunen et al., 2021). Engineered bacteria also enable otherwise challenging mechanistic studies, allowing researchers to learn how individual bacterial functions or proteins in the microbiome contribute to host physiology, pathophysiology, or the microbiome community (Biteen et al., 2016). Although synthetic biologists have designed increasingly elaborate genetic circuits that in principle could risk-stratify patients and combat disease, these circuits have only worked in reduced-community models *in vivo* (i.e., gnotobiotic mice, antibiotic-treated mice) (Claesen and Fischbach, 2015; Mimee et al., 2016).

A key step in engineered LBTs is selection of a microbial host organism, or chassis, which would enable environment-sensing, regulated gene expression, and production of a therapeutic product. Current LBT chassis (e.g., *Escherichia coli* Nissle 1917, *Bacteroides* spp., *Lactobacillus* spp.) cannot engraft or even survive in the gut luminal environment. They require frequent re-administration and are difficult to localize to specific regions, such as the proximal gut, thereby resulting in unreliable delivery of function (Claesen and Fischbach, 2015; Isabella et al., 2018; Pedrolli et al., 2019). The lack of engraftment severely limits the use of LBTs for chronic conditions, for curative effect, or to help devise tools to study specific functions in the gut microbiome (Claesen and Fischbach, 2015; Pedrolli et al., 2019). Published human trials using engineered LBTs demonstrated safety but yielded disappointing physiological results (Braat et al., 2006; Puurunen et al., 2021). The authors attributed therapeutic failure to the inability of the chassis to survive long enough to express the transgene of interest. Hence, appropriate chassis selection remains a significant barrier to translating synthetic biology applications to human diseases.

LBTs face many challenges to survival in the luminal environment, both from the host (e.g., peristalsis, innate and adaptive immunity) and other native microorganisms (e.g., competition, niche availability) (Walker and Owen, 1990). Probiotic strains not adapted to the luminal environment cannot easily compete with microbes that have adapted to their specific luminal niche over thousands of generations (Huang et al., 2021). This phenomenon is apparent in most patients who have received fecal microbiota transplant, where the individual’s native microorganisms return and largely, or completely, displace the transplanted microbes (Li et al., 2016). However, early studies of native *E. coli* strains, such as MP-1 (Lasaro et al., 2014) and NGF-1 (Kotula et al., 2014; Riglar et al., 2017), demonstrate prolonged engraftment in the luminal environment of 70–200 days, respectively, in mice pre-treated with antibiotics and kept in specific pathogen-free (SPF) conditions. More recently, a wastewater *Bacteroides ovatus* strain that was engineered to be tunable to a prebiotic, porphyran, engrafted in the gut with 10^5^–10^12^ colony-forming units (CFUs)/g in the feces of mice housed in gnotobiotic isolators for up to 25 days (Shepherd et al., 2018). However, whether native species, particularly a low-abundance organism such as native *E. coli*, can persistently engraft in non-antibiotic-treated, non-sterile, fully conventional conditions and be used to functionally change the luminal environment, induce physiological change, or treat disease, remains unclear.

In theory, native bacteria are already maximally adapted to the luminal environment of the host, thereby bypassing nearly all the barriers to engraftment, making them an ideal chassis for transgene delivery. The reluctance to use undomesticated native bacteria is driven by the reality that they are difficult to culture and modify, although recent studies demonstrate that they can be modified more consistently with novel methods (Brophy et al., 2018; Jin et al., 2022; Ronda et al., 2019; Rubin et al., 2022; Wiles et al., 2018). Studies in gnotobiotic mice show that a host monocolonized with a single species is resistant to colonization by the same, but not different, species (Lee et al., 2013), suggesting that if the niche of the engineered bacteria is already filled, engraftment would be more difficult. In addition, despite the early success with native strains (Lasaro et al., 2014; Riglar et al., 2017), many have assumed that *E. coli* are not good colonizers due to disappointment with their commensal lab strain or probiotic counterparts (e.g., *E. coli* Nissle 1917). This has led researchers to favor species that are far more abundant in the gut microbiome (e.g., *Bacteroides* spp., *Lactobacillus* spp.). However, these species are much more challenging to engineer. Hence, it may seem that the barriers to using native bacteria as chassis for transgene delivery, particularly for conventionally raised (CR) hosts, are high.

**Figure 1.**
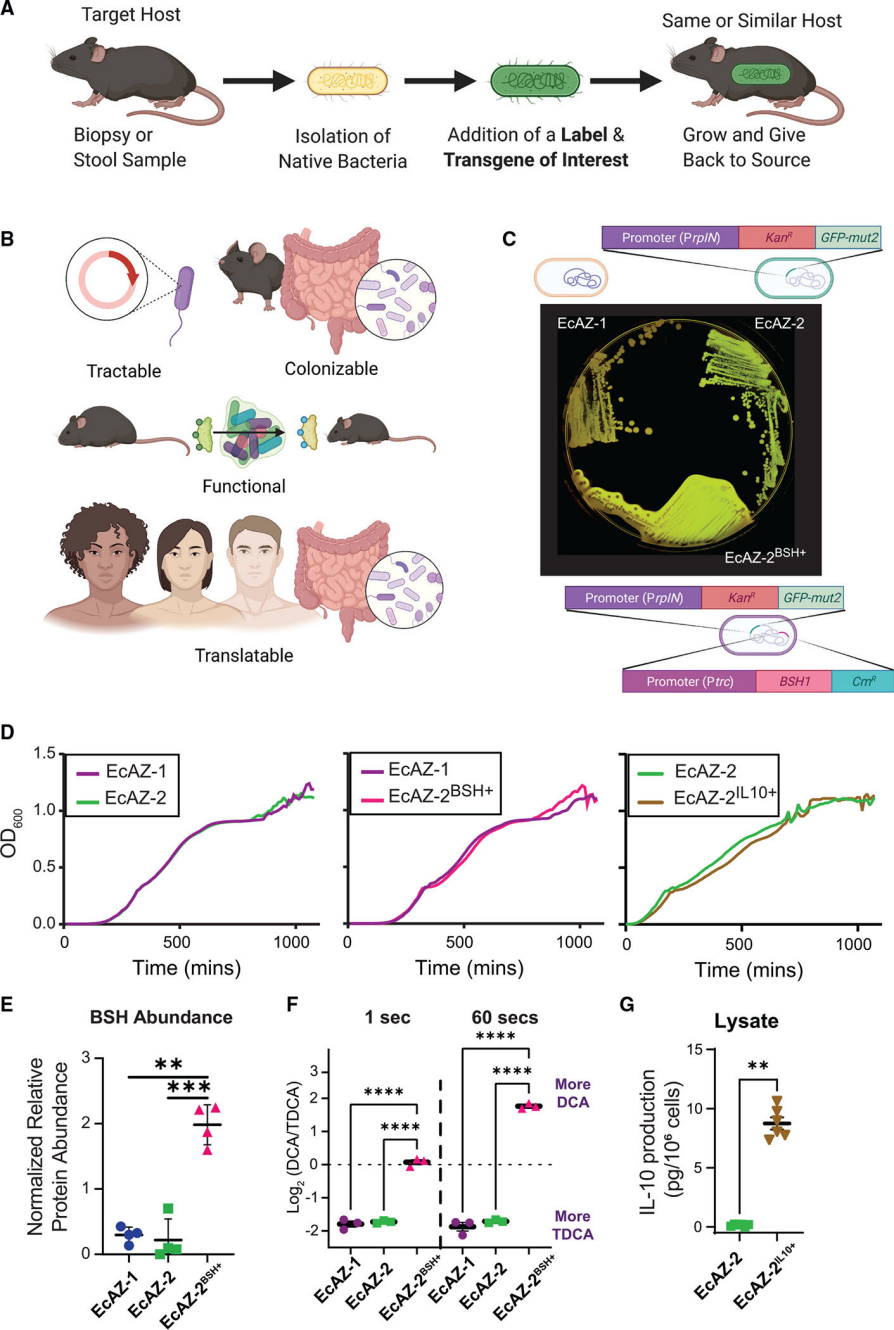
Gut native *E. coli* are genetically tractable and can serve as a chassis for transgene delivery (A) Experimental strategy of engineered native bacteria. (B) Optimal characteristics of a chassis for transgene delivery for a potential LBT. (C) Original isolate (EcAZ-1), GFP-producing strain (EcAZ-2), and GFP- and BSH-producing strain (EcAZ-2^BSH+^) plated on LB containing TDCA. Precipitate around EcAZ-2^BSH+^ is the result of TDCA deconjugated by the bacteria to DCA and qualitatively indicates enzyme functionality. (D) Growth curve of EcAZ-2 compared with EcAZ-1, EcAZ-2^BSH+^ compared with EcAZ-1, and EcAZ-2^IL10+^ compared with EcAZ-2. The line represents an average of three measurements per strain. (E) Proteomic analysis shows increased BSH protein expression in EcAZ-2^BSH+^ compared with EcAZ-1 and EcAZ-2 (n = 4). (F) Log_2_ ratio of DCA to TDCA in a timed enzymatic assay (n = 3; see Figure S1G for raw values). (G) IL-10 levels detected with ELISA from cell lysates (n = 6). The marker covers some error bars in (F) and (G). Given the low number of samples for (E) and (F), we used a Student’s t test after the confirmation of normality with Q-Q plot. We used a Mann-Whitney U test for (G). (A) and (B) were created with BioRender.com. See also Figure S1.

Here we present a proof-of-concept study that advances our ability to use engineered bacteria to effectively change physiology in CR wild-type (WT) hosts by demonstrating that native bacteria can serve as chassis for transgene delivery that modifies the host phenotype. This is accomplished by identifying a genetically tractable, native bacterial strain from a host (i.e., an undomesticated *E. coli*), modifying this strain to express a transgene of interest, and then reintroducing the engineered native bacteria to the host (Figure 1A). The transgenes of interest included bile salt hydrolase (BSH), a prokaryotic bile acid deconjugation enzyme (Figure S1A) that can potentially affect host metabolic homeostasis, and IL-10, a mammalian cytokine that can function as an anti-inflammatory agent. After a single treatment, engineered native bacteria engraft throughout the entire gut of CR hosts for the lifetime of the mouse, retain the function of their transgene, induce metabolomic changes, affect host physiology, and even ameliorate pathophysiological conditions. We evaluate the robustness of the approach by performing experiments in non-sterile facilities, testing biocontainment between co-housed littermates, and changing diets. We demonstrate the translational potential of this method by successfully and stably transforming native, undomesticated, human-derived *E. coli* that can be used for transgene delivery in humans to potentially treat disease. Although the individual steps of the proposed approach for engineering undomesticated native bacteria to express the desired function of interest are not particularly difficult, the combination is novel, and, put together, they clearly demonstrate that even resource-limited labs can accomplish what has yet to be achieved with other synthetic biology approaches: persistent functional manipulation of the luminal environment of CR hosts to study the physiological effects.

## RESULTS

### Gut native *E. coli* bacteria are genetically tractable

An ideal chassis is genetically tractable, can stably and persistently colonize the selected host, express the gene of interest persistently (and without much effect on bacterial fitness), and impose a functional change to alter the lumen and/or serum to affect host physiology (Figure 1B). Importantly, the strategy should be easily translated to humans, as this has been a particular challenge in adapting synthetic biology tools to clinical problems (Figure 1B). In our studies, we used an undomesticated, native *E. coli*, EcAZ-1, isolated from the stool of a CR-WT C57BL/6 male mouse acquired from Jackson Laboratory (Bar Harbor, ME). As measured using MUMmer4 alignments (Març ais et al., 2018), EcAZ-1 is a closely related strain to NGF-1 (Kotula et al., 2014; Riglar et al., 2017), with >99% identity across the full length of the main chromosome and 8-kb plasmid, and 88% in the 42-kb plasmid. *E. coli* MP-1 (Lasaro et al., 2014) is more distantly related, with ~97% identity across only 87% of the EcAZ-1 chromosome. The 8-kb plasmid of EcAZ-1 is present in MP-1 at >99% identity across its length.

Green fluorescent protein (GFP) linked with kanamycin resistance was introduced to EcAZ-1 via phage transduction to create a traceable version of the bacteria, EcAZ-2 (Figures 1C and S1A). EcAZ-2 was then engineered, separately, to form two different engineered native bacteria. The first, EcAZ-2^BSH+^, expresses BSH (Figures S1B and S1C), a bacterial gene that causes specific bile acid biotransformations demonstrated to affect the host metabolic phenotype in reduced-community microbiome studies (e.g., gnotobiotic mice, antibiotic-treated mice) (Jones et al., 2008; Joyce et al., 2014a; Yao et al., 2018). *In vitro* BSH activity was monitored with the aid of taurodeoxycholic acid (TDCA) plates; as EcAZ-2^BSH+^ deconjugates TDCA to deoxycholic acid (DCA), these plates form a white precipitate around the bacterial colony (Figure 1C). The second gene, IL-10 (to form EcAZ-2^IL10+^; Figure S1D), is a mammalian anti-inflammatory cytokine. Although continuous administration of IL-10-expressing probiotics has been successful in treating preclinical models of colitis (Steidler et al., 2000), this therapy has not successfully translated to humans, perhaps due the inability of chassis to survive in the luminal environment (Braat et al., 2006). The addition of *gfp* and kanamycin resistance, as well as *bsh* and *Il-10*, did not affect the *in vitro* growth rate of EcAZ-2, EcAZ-2^BSH+^, and EcAZ-2^IL10+^ (Figure 1D; Figure S1E). Sequencing of EcAZ-1 and EcAZ-2 demonstrates that these two bacteria remain almost identical. Other than the deliberate engineering, there are no other indels and only ~4,500 SNPs, which is in the range of the error rate of Illumina sequencers. The same is true for EcAZ-2^BSH+^, with fewer than ~10,000 SNPs versus EcAZ-1 and ~4,000 versus EcAZ-2, and no unexpected indels. Thus, native *E. coli* are genetically tractable, can express prokaryotic and eukaryotic transgenes, and can potentially serve as chassis for transgene delivery.

We performed additional assays to quantitatively assess protein expression in EcAZ-2^BSH+^ and EcAZ-2^IL10+^. Proteomic analysis demonstrates that BSH is one of the most highly expressed proteins in EcAZ-2^BSH+^, whereas it is not detectable in EcAZ-1 or EcAZ-2 (Figure 1E; Figure S1F). Constitutively expressed BSH produced by EcAZ-2^BSH+^ converts TDCA to DCA in a controlled *in vitro* assay rapidly, whereas no shift is seen with EcAZ-1 or EcAZ-2 (Figure 1F; Figure S1G). For EcAZ-2^IL10+^, IL-10 was detected in cell lysates but not the supernatant (Figure 1G). Thus, proteins produced by native *E. coli* are functional and highly expressed constitutively.

### Engineered native *E. coli* can engraft in the gut lumen

**Figure 2.**
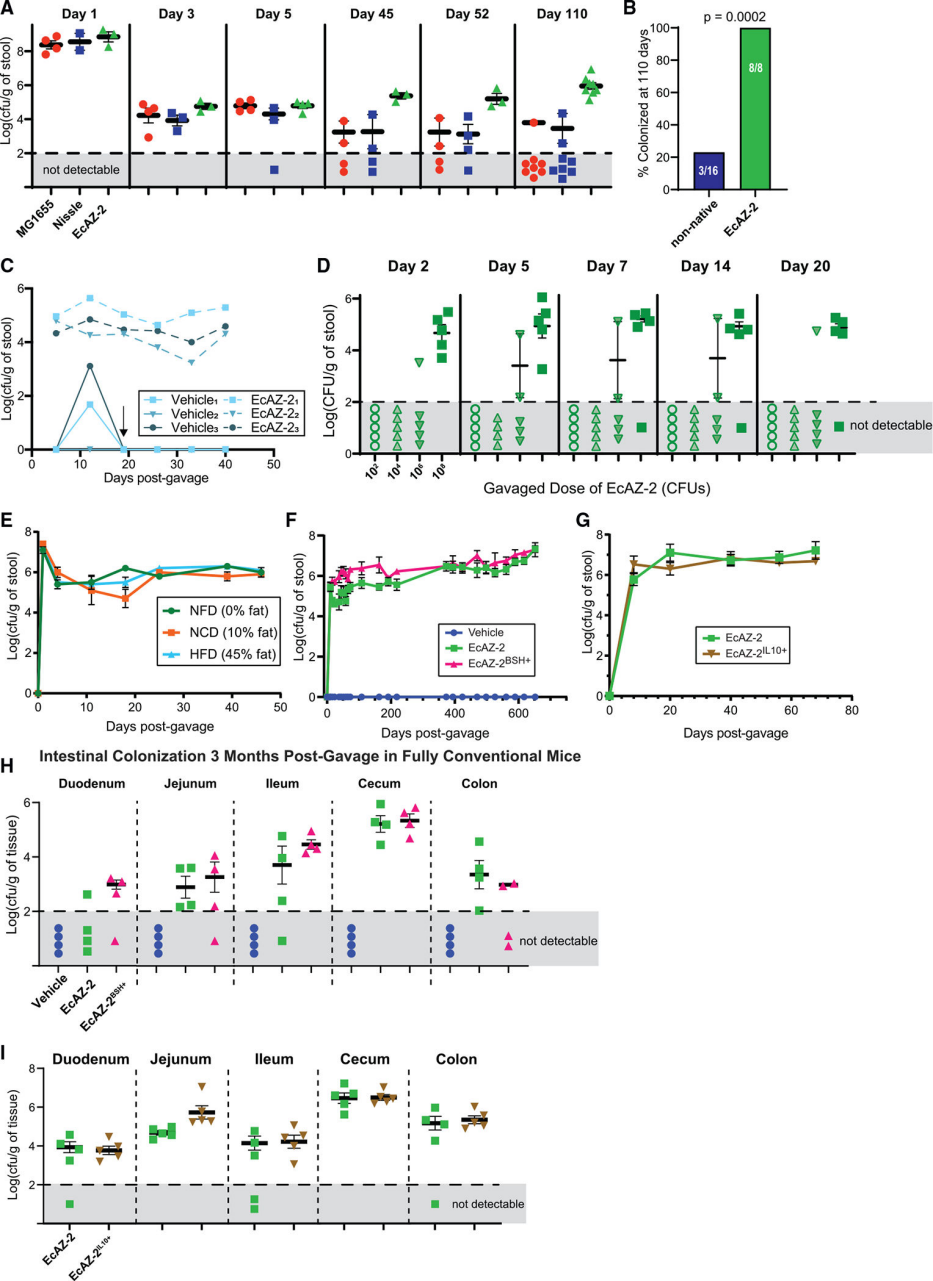
Engineered native *E. coli* can engraft in the luminal environment (A) Long-term colonization after gavage with 10^10^ CFUs of E. coli MG1655 and *E. coli* Nissle 1917 (two lab-strain *E. coli* often used as chassis for function delivery), as well as EcAZ-2 in non-antibiotic-treated CR-WT mice housed in a low-barrier, non-sterile facility after a single gavage. Sample measures in the gray area denote mice where the bacteria were not detectable at a limit of detection of Log_10_ of 2. (B) Colonization at 110 days (Fisher’s exact test; 4–8 mice/condition). (C) Colonization in co-housed mice housed in a low-barrier, non-sterile facility after a single gavage, where one mouse received 10^10^ CFUs of EcAZ-2 and the other received vehicle via oral gavage. Arrow indicates change in stool collection protocol where mice were separated from their cage mates 24 h prior to their weekly stool collection. Mice were returned to their cage mates right after stool collection. Thus, although uncolonized mice are exposed to EcAZ-2 through coprophagia, the level is not sufficient to lead to engraftment. (D) Colonization after gavage of different initial doses of EcAZ-2 in non-antibiotic-treated CR-WT mice housed in a low-barrier, non-sterile facility after a singlegavage (n = 5). (E) Colonization after gavage of 10^10^ CFUs of EcAZ-2 in the stool of non-antibiotic-treated CR-WT mice in an SPF facility on a stable diet (10% fat) or on diets with different macronutrient profiles (non-fat diet [NFD] or high-fat diet [HFD]). Diet was changed 4 days post-gavage (4 mice/condition). (F) Colonization after gavage of 10^10^ CFUs of EcAZ-2 or EcAZ-2^BSH+^ in non-antibiotic-treated CR-WT mice housed in an SPF facility (4–12 mice/condition). (G) Colonization after gavage of 10^10^ CFUs of EcAZ-2 or EcAZ-2^IL10+^ in non-antibiotic-treated CR-WT mice housed in an SPF facility (4 mice/condition). (H) Colonization of gastrointestinal tract in CR-WT mice 3 months after a single gavage in an SPF facility. Sample measures in the gray area denote mice where the bacteria were not detectable at a limit of detection of Log_10_ 2. (4 mice/condition.) (I) Colonization of EcAZ-2 and EcAZ-2^IL10+^ in the gastrointestinal tract of CR-WT mice housed in an SPF facility 79 days after gavage (same mice depicted in Figure 2E; 5/condition). All error bars indicate standard error of the mean. Samples that were not detectable were excluded from mean and standard error of mean calculations. The marker covers some error bars in (E), (F), and (G). See also Figure S2.

Next, we assessed the candidate chassis’ ability to stably colonize the murine gut by gavaging 10^10^ CFUs of engineered bacteria with different *E. coli* chassis in CR-WT C57BL/6, non-antibiotic-treated mice. EcAZ-2 stably maintained colonization in 100% (n = 8) of mice for over 110 days in a non-sterile, low-barrier facility (Figures 2A and S2A). Domesticated lab bacteria that are often used as chassis for LBT applications (i.e., *E. coli* MG1655 and *E. coli* Nissle 1917), lost colonization over time (p = 0.02, log rank test, Figure S2A) and did not maintain colonization long-term (p < 0.0001, Fisher’s exact test, Figure 2B). Mice that had detectable non-native *E. coli* in their stool were nearly two orders of magnitude lower (as determined by CFU/g of stool) than those treated with EcAZ-2 (Figure 2A). Co-housing of mice colonized with EcAZ-2 with non-colonized mice did not lead to horizontal transmission between mice, despite the opportunity for coprophagia (Figure 2C). This is likely due to an insufficiently high enough dose of bacteria being transmitted through coprophagia, as gavage with 10^2^ and 10^4^ CFUs of EcAZ-2 in CR-WT C57BL/6, non-antibiotic-treated mice did not lead to engraftment in any mice in the non-sterile low-barrier facility, whereas 10^6^ CFUs led to sporadic—and 10^8^ CFUs more consistent—engraftment (Figure 2D). Mice gavaged with 10^8^ CFUs were significantly more likely to be colonized than those gavaged with lower doses at every time point measured (Fisher’s exact test, Figure S2B).

Colonization of EcAZ-2 was unaffected by changes to the diet macronutrient profile (Figure 2E). The addition of BSH or IL-10 activity does not significantly alter the colonization level of EcAZ-2; this native strain remains stably colonized in CR-WT mice for the life of the animal, in reproducible experiments (Figures 2F and S2C, for BSH; Figures 2G and S2D, for IL-10) in both SPF and non-sterile facilities. Sex of the CR-WT host did not affect how well EcAZ-2 colonized the host (Figure S2E).

To determine whether BSH function was retained during engraftment, bacterial isolates from mice treated with EcAZ-2^BSH+^ were grown on TDCA plates. Isolates from every region of the gastrointestinal (GI) tract of every mouse treated with EcAZ-2^BSH+^, across every experiment, retained BSH functionality, confirmed by deconjugation of TDCA to DCA (see Figure S2F). Likewise, cell lysates of bacterial isolates obtained from mice treated with EcAZ-2^IL10+^ at 89 days after gavage produced IL-10 at far higher levels than the negative control bacteria (i.e., EcAZ-2; Figure S2G).

We cultured gastrointestinal tissues (i.e., duodenum, jejunum, ileum, cecum, and rectum) to determine the extent of bacterial colonization in the various regions of the gut. In contrast to what has been reported for engineered non-native *E. coli* (Isabella et al., 2018), EcAZ-2 colonizes the proximal gut/small intestine regardless of whether it contains the *bsh* gene, *Il-10* gene, or not, with the highest colonization in the cecum and ileum for the BSH strain and cecum and jejunum for the IL-10 strain (Figures 2H, 2I, and S2H). Colonization throughout the GI tract was consistent in fed and fasted mice (Figure S2H), and isolates from throughout the GI tract retained transgene activity (Figure S2I). These experiments demonstrate that EcAZ-2 can stably and persistently colonize CR-WT hosts for perpetuity, while retaining functionality of a gene of interest.

### Engineered native *E. coli* can perform a luminal function

**Figure 3.**
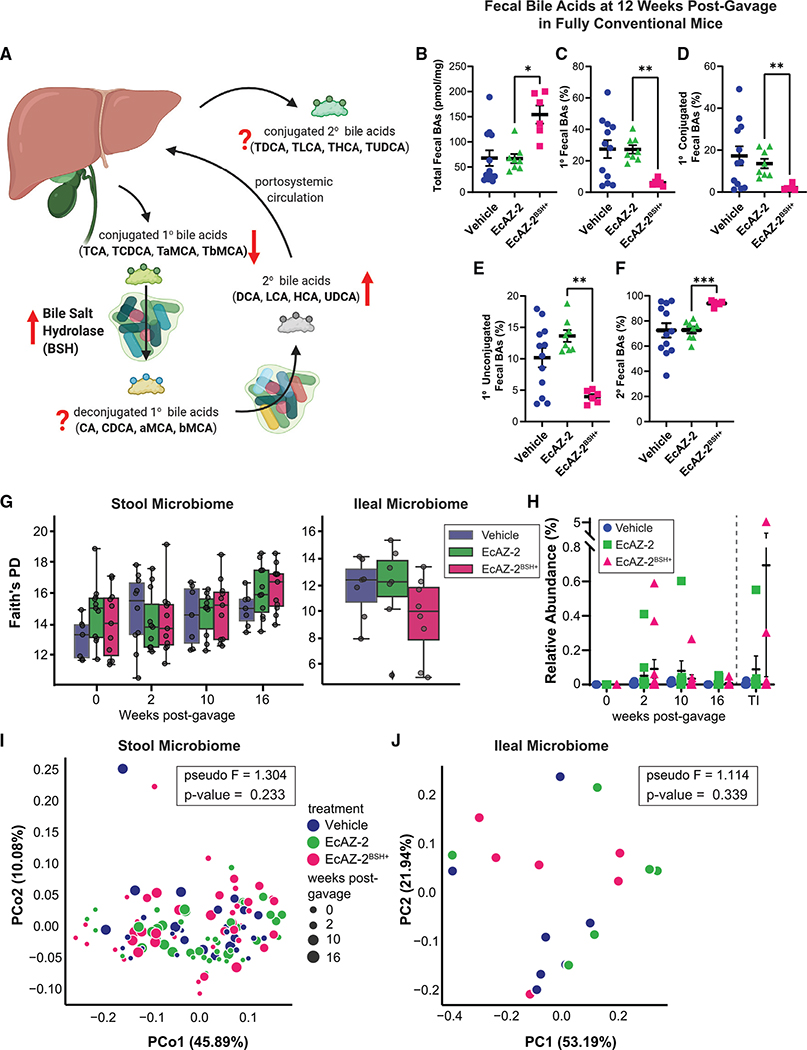
Native *E. coli* can be used to change luminal metabolome without measurable effects in the microbiome (A) Summary diagram showing effects of BSH on fecal bile acid metabolism. The hypothesized effect of increased BSH on the different bile acid pools is shown with red arrows. A question mark denotes uncertainty. (B–F) (B) Total fecal bile acids, (C) primary bile acids, (D) primary conjugated bile acids, (E) primary unconjugated bile acids, and (F) secondary bile acids in fecal samples collected from mice 12 weeks after a single gavage with vehicle, EcAZ-2, and EcAZ-2^BSH+^. Significant differences were determined by Kruskal-Wallis test with post hoc Dunn’s multiple comparison test comparing EcAZ-2 and EcAZ-2^BSH+^. (G) Faith’s phylogenetic distance (a measure of α-diversity) from 16S rRNA gene sequencing performed on stool samples collected pre-treatment and at 2, 10, and 16 weeks post-gavage (left) and from the terminal ileum samples at the time of euthanasia (right). (H) Relative abundance of *E. coli* as detected by 16S rRNA gene sequencing performed on stool samples collected pre-treatment and at 2, 10, and 16 weeks post-gavage and from the terminal ileum samples at the time of euthanasia. (I) Principal coordinate analysis of weighted UniFrac β-diversity of the fecal samples collected pre-treatment and at 2, 10, and 16 weeks post-gavage. (J) Principal coordinate analysis of weighted UniFrac β-diversity of the terminal ileum microbiome at the time of euthanasia. (A) was created with BioRender.com. See also Figure S3.

After demonstrating that native bacteria are genetically tractable and can persistently colonize the proximal gut and colon, we assessed whether the transgene delivered by the EcAZ-2 chassis can functionally manipulate the luminal environment of CR hosts. We used *bsh* as our transgene to evaluate the native bacterial chassis because its functions are well-described and it has been studied in reduced-community/gnotobiotic models (Jones et al., 2008, 2014a; Yao et al., 2018). Bile acid changes induced by BSH activity are presumed to affect multiple metabolic systems, including glucose, lipid, and bile acid homeostasis, as well as immunity/inflammation and thermogenesis (Joyce et al., 2014b; Wahlström et al., 2016). Hepatic primary conjugated bile acids are deconjugated by BSH (Figure 3A). Whereas the ileum has active transporters for the highly polar conjugated bile acids, deconjugated bile acids are less polar and are reabsorbed through passive diffusion. In the distal gut, deconjugated bile acids are substrates for luminal bacterial bile acid biotransformations that convert them to secondary bile acids. Secondary bile acids can be reabsorbed by passive diffusion, conjugated by the liver into conjugated secondary bile acids, and re-released into the lumen. Hence, increased BSH activity can affect all of these various bile acid pools.

To confirm that BSH-expressing native *E. coli* are functional *in vivo*, we determined the effect of EcAZ-2^BSH+^ on the bile acids of mono-colonized gnotobiotic mice. Whereas mice mono-colonized with EcAZ-2 had only primary conjugated bile acids and no deconjugated bile acids (because they lack bacteria that can deconjugate), mice mono-colonized with EcAZ-2^BSH+^ had greatly reduced primary conjugated fecal bile acids (Figure S3A) and increased primary deconjugated fecal bile acids (Figure S3B), consistent with increased BSH activity. Quantitative analysis of the conjugated and unconjugated individual primary bile acids showed that EcAZ-2^BSH+^ significantly shifted the log ratio of cholic acid to taurocholic acid (CA:TCA), and β-muricholic acid to tauro-β-muricholic acid (bMCA:TbMCA), toward the deconjugated bile acids in mono-colonized mice (Figure S3C).

To determine whether engineered native bacteria expressing BSH can functionally manipulate the fully intact microbiome of CR mice, we gavaged EcAZ-2 or EcAZ-2^BSH+^ into 11-week-old CR-WT mice. 12 weeks after the single oral administration, stool samples were collected and analyzed for bile acids. CR mice colonized with EcAZ-2^BSH+^ had significantly higher total fecal bile acid levels than CR mice colonized with EcAZ-2 (Figure 3B). In addition, CR mice gavaged with EcAZ-2^BSH+^ had decreased primary fecal bile acids (Figure 3C) and decreased primary conjugated bile acids (Figure 3D) compared with mice treated with EcAZ-2. Surprisingly, primary unconjugated bile acids were also significantly decreased in EcAZ-2^BSH+^-treated mice compared with EcAZ-2 controls (Figure 3E). However, this could be due to increased conversion of deconjugated bile acids to secondary bile acids, which was significantly elevated in EcAZ-2^BSH+^ mice (Figure 3F). In fact, treatment with EcAZ-2^BSH+^ affected nearly every fecal bile acid measured, including TbMCA, a potent farnesoid X receptor (FXR) antagonist, and DCA, a potent G protein-coupled bile acid receptor 1 (TGR5) agonist (Figure S3D; Wahlström et al., 2016). These fecal bile acid data confirm that BSH is functional *in vivo*. Moreover, they demonstrate that native *E. coli* are valuable chassis that can express a function of interest intraluminally at least 12 weeks after a single gavage in CR-WT mice.

We expected that changes in intraluminal bile acids would lead to global changes in gut microbiome composition. However, microbiome analysis showed that EcAZ-2 and EcAZ-2^BSH+^ had no detectable effect in any microbiome measures. Compared with mice treated with vehicle, we found no significant differences in Faith’s phylogenetic diversity in the fecal microbiome at 2, 10, and 16 weeks post-gavage or in the ileum after euthanasia (Figure 3G). Other measures of α-diversity such as richness (Figure S3E) and the Shannon index (Figure S3F) found a transient difference at week 2 between mice in the three conditions that disappeared by the later time points. Differential abundance analysis using ALDEx2 (Fernandes et al., 2014) found no compositional differences at significance (p ≤ 0.05), even before any type of multiple hypothesis correction between EcAZ-2 and vehicle, either with or without BSH, or any between EcAZ-2 and EcAZ-2^BSH+^, either across all samples or at any specific time point alone. Confirmatory analysis with ANCOM (Mandal et al., 2015) demonstrated no compositional difference between EcAZ-2 and EcAZ-2^BSH+^, and only two amplicon sequence variants (ASVs) between EcAZ-2 and vehicle (assigned as Lachnospiraceae and Bacteroidaceae), and one between EcAZ-2^BSH+^ and vehicle (assigned as Clostridiacae) that was inconsistent across time points. Hence, despite inducing changes in the luminal metabolome, engineered native bacteria in this case did not induce a compositional shift in the gut microbiome.

There were no *E. coli* detectable by ASV matching the 16S rRNA gene sequence of *E. coli* prior to gavage. Although *E. coli* could be identified in some of the samples from mice treated with EcAZ-2 or EcAZ-2^BSH+^, most had levels of *E. coli* in stool that were statistically indistinguishable as a group from the vehicle-treated controls (Figure 3H). Overall, based on 16S rRNA gene sequencing, *E. coli* comprised <0.1% of the fecal microbiome. Principal coordinate analysis of the UniFrac weighted β-diversity performed at 2, 10, and 16 weeks could not distinguish the three treatment groups (Figure 3I; pseudo-F = 1.304, p = 0.233). The lack of a difference is reflected in the ileum as well (Figure 3J; pseudo-F = 1.114, p = 0.339). This demonstrates that, even at low abundances, the native *E. coli* can be used to introduce a function of interest in CR hosts, and that they do so without significantly altering the microbial community already established in the host.

### Engineered native *E. coli* bacteria affect host physiology

**Figure 4.**
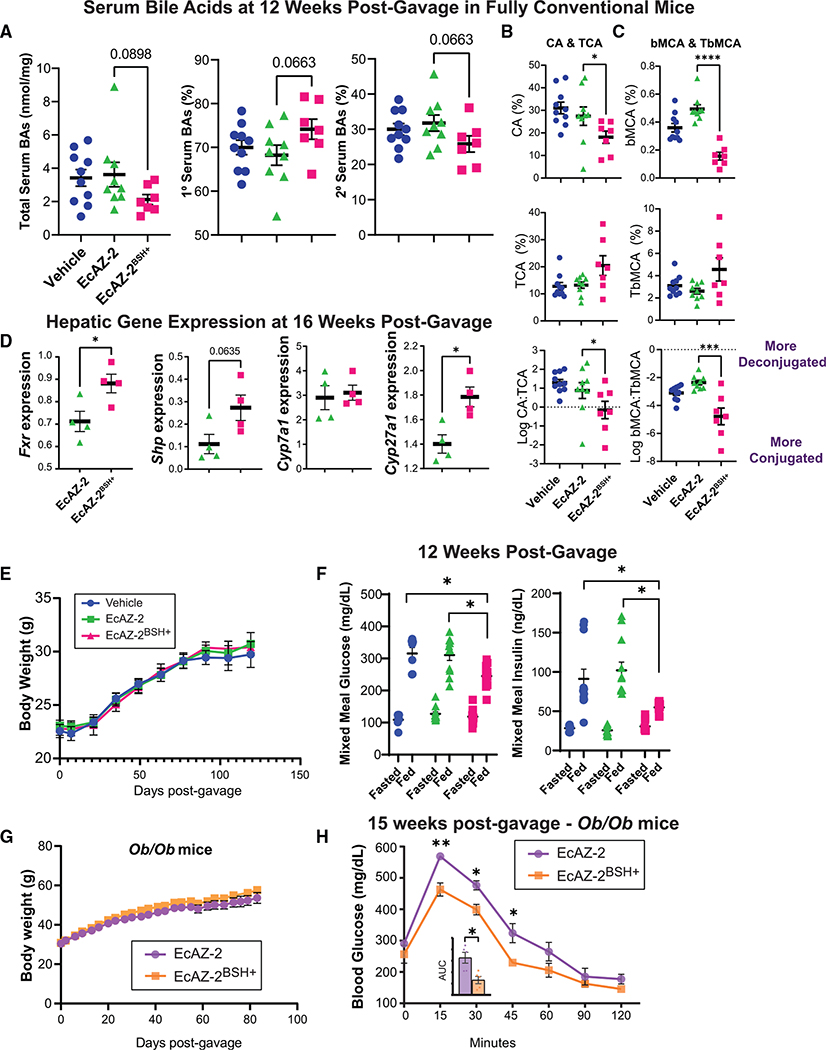
Native *E. coli* can be used to physiologically change the host and treat pathophysiological conditions (A) Total, primary, and secondary serum bile acids from mice treated with a single gavage of vehicle, EcAZ-2, or EcAZ-2^BSH+^ 12 weeks prior. (B) Serum levels of CA (top), TCA (middle), and the log_2_ ratio of CA to TCA in mice treated with vehicle, EcAZ-2, or EcAZ-2^BSH+^. (C) Serum levels of bMCA (top), TbMCA (middle), and the log_2_ ratio of bMCA to TbMCA in mice treated with vehicle, EcAZ-2, or EcAZ-2^BSH+^. (D) Hepatic gene expression of *Fxr*, *Shp*, *Cyp7a1*, and *Cyp27a1*, as determined by qRT-PCR. Significant differences were determined by a Student’s t test after normality verified through Q-Q plot. (E) Mouse weights of CR-WT mice treated with a single gavage of vehicle, EcAZ-2, and EcAZ-2^BSH+^. (F) Fasting (16 h) and postprandial (30 min) glucose and insulin levels. Significant differences were determined by Kruskal-Wallis test with post hoc Dunn’s multiple comparison test comparing all three conditions done separately for fasted and fed measures. (G) Mouse weights of *Ob/Ob* mice treated with a single gavage of EcAZ-2 and EcAZ-2^BSH+^. (H) Glucose tolerance test in *Ob/Ob* mice treated with a single gavage of EcAZ-2 and EcAZ-2^BSH+^ 15 weeks prior. Significance determined with Mann-Whitney U test corrected for multiple comparisons. Inset shows area under the curve. Significance determined by Mann-Whitney U test. For (A), (B), and (C), significant differences were determined by a Kruskal-Wallis test with post hoc Dunn’s multiple comparison test comparing EcAZ-2 and EcAZ-2^BSH+^. See also Figure S4.

We next investigated whether transgene delivery and expression by the native *E. coli* chassis can affect host physiology by first determining the impact of colonization with EcAZ-2^BSH+^ on circulating bile acids. Mice colonized with EcAZ-2^BSH+^ had reduced total serum bile acids, increased primary bile acids, and decreased secondary bile acids, though these did not achieve significance (Figures 4A and S4A). There were larger changes in specific circulating bile acids, including known agonists and antagonists of FXR, such as increased TCA and TbMCA, respectively, compared with their deconjugated counterparts (Figures 4B, 4C, and S4B). We measured the impact of these differences in circulating bile acids on hepatic gene expression, focusing on genes important for bile acid metabolism. We found increased expression of *Fxr*, *Shp*, and *Cyp27a1* (Figure 4D), consistent with increased bile acid biosynthesis through the alternative pathway, perhaps to alleviate the higher excretion of bile acids in the stool of mice colonized with EcAZ-2^BSH+^. This demonstrates that the addition of a single gene into the microbiome via engineered native bacteria can significantly alter host physiology.

To determine whether the transgene delivery with the native *E. coli* chassis can induce long-lasting change in host physiological phenotype, we metabolically characterized the cohort of mice that received vehicle, EcAZ-2, or EcAZ-2^BSH+^ approximately 12 weeks after gavage. Unlike what has been reported in gnotobiotic and reduced-community mice (Joyce et al., 2014a; Yao et al., 2018), increased BSH activity induced with engineered native bacteria did not affect the body weight of the host (Figure 4E). The addition of EcAZ-2 or EcAZ-2^BSH+^ did not affect food intake (Figure S4C). 12 weeks after a single gavage with engineered native bacteria, CR mice on a normal chow diet colonized with EcAZ-2^BSH+^ had reduced postprandial blood glucose and insulin levels compared with those colonized with EcAZ-2 after a mixed meal challenge (Figure 4F). We repeated the mixed meal challenge in a separate experiment using female mice with a shorter duration of colonization with engineered native bacteria (Figure S4D). As with the male mice, the female mice treated with EcAZ-2^BSH+^ had lower postprandial insulin levels (Figure S4E).

Decreased postprandial insulin induced by EcAZ-2^BSH+^ suggests improved insulin sensitivity and glucose tolerance. To determine whether transgene delivery with a native *E. coli* chassis could be used to ameliorate disease, we investigated the effect of EcAZ-2^BSH+^ in a genetic model of type 2 diabetes, the *Ob/Ob* mice. EcAZ-2 and EcAZ-2^BSH+^ stably colonized CR *Ob/Ob* mice (Figure S4F). Similar to what we had observed in CR-WT mice, EcAZ-2^BSH+^ did not induce a change in body weight (Figure 4G). Despite identical body weights, *Ob/Ob* mice colonized with EcAZ-2^BSH+^ had significant improvements in insulin sensitivity compared with the control cohort (Figure 4H) after receiving a single treatment of engineered native bacteria more than 3 months earlier. Thus, these experiments show that transgene delivery using native *E. coli* affects the serum metabolome, extra-intestinal (e.g., hepatic) gene expression, and host physiology, and ameliorates disease months after a single administration.

### Humans have genetically tractable native *E. coli*

To determine whether the engineered native bacteria strategy can be translated to humans, we isolated *E. coli* strains from biopsies obtained from multiple regions of the gastrointestinal tracts of volunteers undergoing routine outpatient endoscopy. Nine isolated strains were assessed for antibiotic sensitivity, tractability via transduction, transformation with assorted sizes of plasmids, and conjugation (a subset shown in Table 1). Many isolates were sensitive to commonly used laboratory antibiotics, including kanamycin, chloramphenicol, and carbenicillin. All isolates evaluated were resistant to transduction via P1*vir* when lysates were generated from *E. coli* MG1655 or *E. coli* Nissle 1917. Strains were transformable with several differently sized plasmids. Conjugation using a modified F-plasmid (Figure S5) proved to be a universally successful method for manipulation for 10/10 human-derived *E. coli*, quantified for a subset of the strains shown in Table 1. To encourage plasmid retention, conjugation machinery was removed post-transfer. Thus, the first steps of the engineered native bacteria approach, identification/isolation of genetically tractable bacteria and modification to express a gene of interest, can be translated to humans. Further studies are necessary to determine whether the autologous transfer of engineered native bacteria can lead to long-lasting colonization of a human host and treatment of long-standing chronic conditions and genetic diseases.

## DISCUSSION

Most therapies that target microbiome composition do not have a detectable impact on the gut microbiome and are not robust to the interpersonal diversity and plasticity of the microbiome in human hosts (Kristensen et al., 2016; Mimee et al., 2016). In addition, many different configurations of the gut microbiota lead to the same functional result (Human Microbiome Project, 2012; Turnbaugh et al., 2009), suggesting that microbial function may be difficult to manipulate with therapies that target the composition of the microbiome alone. To develop a better mechanistic understanding of the microbe-host relationship and more effective microbiome-mediated therapies, approaches based on functional modulation of the gut microbiome are necessary. However, these approaches have been difficult to develop, and none have demonstrated long-term engraftment with a change in physiology and/or improvement of pathologic phenotype.

To address the inability of engineered bacteria to colonize the CR host, only three strategies have shown success (Bober et al., 2018; Claesen and Fischbach, 2015; Mimee et al., 2016; Riglar and Silver, 2018; Sheth et al., 2016): using native *E. coli* as chassis (Kotula et al., 2014; Lasaro et al., 2014; Riglar et al., 2017); creating exclusive metabolic niches for an engineered probiotic strain, which can then outcompete resident microorganisms (Shepherd et al., 2018); and engineering non-colonizing bacteria to distribute mobile genetic elements (e.g., plasmids) to native bacteria *in vivo* (Ronda et al., 2019). However, none of these approaches have been demonstrated to engraft in the proximal gut, achieve engraftment in non-sterile facilities, alter luminal or serum metabolome, affect host physiology, or demonstrably ameliorate disease, as shown here.

A promising approach has been to find genes that improve engraftment in commonly used chassis, such as *E. coli* Nissle 1917 (Crook et al., 2019). More commonly, investigators have been developing tools to manipulate families of bacteria that are found in large numbers in the gut microbiome to determine whether they can outcompete or coexist with native strains and engraft in the intestinal lumen (Bermúdez-Humarán et al., 2013; Guglielmetti et al., 2013; Lim et al., 2017; Whitaker et al., 2017). Of particular interest has been Bacteroides spp., including *B. ovatus* and *B. thetaiotaomicron* (Lim et al., 2017; Mimee et al., 2015; Shepherd et al., 2018). These bacteria and other engineered probiotics have been exclusively assessed under reduced-community (as opposed to CR) conditions (i.e., gnotobiotic mice, antibiotic-treated mice) and have not yet demonstrated whether they can be used to alter the metabolome and host physiology, and reverse pathology in fully conventional mice. Moreover, these efforts have advanced our understanding of bacterial engraftment, biogeography of microorganisms and luminal micro-niches, and the biological requirements for bacterial colonization and function delivery. However, they also indicate that the amount of bacterial engineering and experimentation necessary to develop chassis that can colonize the gut is daunting and out of the reach for resource-limited labs without dedicated synthetic biologists. Moreover, they are problematic for studying steady-state mechanisms in the gut, unlikely to work in animal models where diet and host genetics are manipulated, and limit feasibility of these techniques for adoption and interventions in humans.

Here, we demonstrate a simple technique that advances our ability to use engineered bacteria to effectively change physiology in CR-WT hosts, using tools and approaches that can be adopted by nearly any lab. By combining existing techniques to native strains, we demonstrate that undomesticated *E. coli* derived from the host can be used as chassis to knockin specific functions into the gut microbiome, thus making mechanistic studies of the gut microbiome attainable by most labs without advanced synthetic biology experience. *E. coli* are easily culturable, common in the human gut (Bailey et al., 2010; Bok et al., 2018; Li et al., 2010; Najjuka et al., 2016; Stoppe et al., 2017), and tools to engineer them have existed for decades. Previous studies have also demonstrated that native bacteria are tractable and can engraft, although these studies were not focused on affecting host physiology or reversing pathology (Lasaro et al., 2014; Riglar et al., 2017). In this study, we show that by using native *E. coli* as our chassis, the resultant engineered native bacteria can: (1) stably colonize CR-WT hosts for months, possibly in perpetuity after a single gavage, without the need for pre-treatment for microbiome depletion; (2) engraft the proximal gut in addition to the colon; (3) demonstrate natural biocontainment between co-housed mice; and (4) alter specific luminal metabolites as intended, that leads to (5) a change in serum metabolites, which then (6) change host metabolic homeostasis and core physiological processes, and thus (7) prevent dysmetabolic phenotypes. Moreover, our preliminary data show that the initial steps of this process can be translated to humans, where we have found readily tractable, native *E. coli* that could be transformed to express a gene. Importantly, we show that identical cohorts that only differ in a single prokaryotic gene in a single low-abundance bacterial strain can have a dramatically different metabolic phenotype. Although this has been demonstrated with pathogenicity islands (e.g., Shiga toxin), we now demonstrate that a beneficial bacterial gene can also have systemic effects.

Earlier studies of an *E. coli* strain isolated from mouse fecal samples from a different facility (i.e., NGF-1) (Lasaro et al., 2014; Ziesack et al., 2018) likewise demonstrated long-term colonization (Riglar et al., 2017). However, it was not clear whether undomesticated *E. coli* strains could be used to study specific bacterial functions, affect the luminal environment, and shape host physiology. Given that *E. coli* abundance is <0.1% of the microbiome, others have focused on chassis that belong to more highly abundant strains. Interestingly, our study shows that colonization of the host luminal environment with our engineered *E. coli* did not affect the overall composition of the microbiome nor the relative abundance of *E. coli*, as detected by 16S rRNA gene sequencing analysis. This suggests several possibilities, including that (1) *E. coli* potentially occupies a particular niche that enables close interaction with the host without too much change in the overall composition of the microbiome, and that (2) *E. coli* is replacing or co-existing with its clonal relatives. Large microbiome resource studies, such as the Human Microbiome Project and American Gut Project (Lloyd-Price et al., 2019; McDonald et al., 2018), show limited detection of *E. coli* by 16S rRNA gene sequencing from fecal samples, whereas infants have higher levels (Chu et al., 2017), suggesting that *E. coli* abundance may be affected by niche availability. Moreover, low-abundance individual microbes can have large ecosystem effects because of the niche they occupy and the functions they perform (Banerjee et al., 2018); thus, a microbe need not be abundant to have a big impact. Overall, our ability to find genetically tractable *E. coli* in humans suggests that this approach can be translated to patients.

Our results clearly demonstrate that native *E. coli* are particularly adept at affecting host physiology and, considering the engineering toolbox that has been developed to genetically manipulate them, *E. coli* make an effective chassis. In addition, having chassis that are unable to engraft has been proposed as a mechanism for biocontainment as it would improve the safety profile by removing concerns about LBT proliferation, transmission to healthy cohabitants, and therapy delivery beyond the end of treatment (Claesen and Fischbach, 2015; Isabella et al., 2018). However, given the inability of current developed methods to effect change in CR hosts, including humans, the development of techniques to make transgene expression tunable (Lim et al., 2017; Whitaker et al., 2017), and our results showing biocontainment with co-housed mice, we suggest that transgene delivery with engineered native chassis will be a far more effective approach.

The technical capability to change microbiome function in CR mice is critical to understanding the role of the gut microbiome in various physiological and pathophysiological processes. Many animal models of disease cannot be recapitulated in reduced microbiome community conditions. For example, baseline metabolic activity in microbiome-depleted (e.g., gnotobiotic, antibiotic-treated) mice is vastly different from that in CR mice (Wichmann et al., 2013; Zarrinpar et al., 2018). This could be why the effects of BSH on host metabolic physiology have been difficult to understand, as prior studies using different reduced-community models have led to contradictory results (Joyce et al., 2014a; Yao et al., 2018). Though BSH is a prevalent function in the luminal environment, it has been identified as an important bacterial function in metabolic homeostasis and immunity (Dantas Machado et al., 2022; Joyce et al., 2014b; Wahlström et al., 2016), making it an ideal first bacterial function to evaluate. We selected our particular BSH (from the strain *Lactobacillus salivarius*) due to its promiscuity and effectiveness (Jones et al., 2008). However, we acknowledge that BSH from other strains could have no effect, or a different effect, on host physiology than what we have shown. Hence, functional manipulation of the microbiome should be paired with other omics analysis (e.g., metabolomics, host transcriptomics) to better understand the host-microbe relationship.

Because deconjugated bile acids do not have dedicated transporters and require reabsorption through passive diffusion, increased luminal BSH activity should lead to an increase in fecal excretion of bile acids, though this has not been reported in microbiome-depleted models (Joyce et al., 2014a). Moreover, they ignore the downstream compositional, metabolomic, and proteomic effects that can lead to changes to the luminal community that could amplify or dampen the effects of the knocked-in function. For example, though increased BSH activity is hypothesized to lead to an increase in primary unconjugated bile acids, we observed the opposite. Instead, because deconjugated bile acids are substrates that some bacteria can convert to secondary bile acids, we observed an increase in secondary bile acids. This observation is apparent in CR mice but not in mono-colonized or reduced-community mice. Although our present work focuses on providing a proof-of-concept study, the BSH results show something different from reduced-community studies—a decrease in serum insulin in the setting of unchanged body weight—which profoundly changes our understanding of how microbiome bile acid biotransformations affect host metabolism (Joyce et al., 2014b). It is unclear why bile acids are such powerful microbial signaling agents. Unlike amino acids and carbohydrates, which are shuttled to the liver through the portal circulation, medium- and long-chain fatty acids bypass the liver because they are absorbed through the lymphatic system (Ticho et al., 2019; Xiao et al., 2019). Hence, bacterially modified bile acids may serve as a nutrient signal that informs the liver about luminal fatty acid absorption. This hypothesis warrants further investigation with dedicated experiments, which can now be tested with engineered native bacteria.

Many investigators may favor using non-engrafting species, such as food-grade *L. lactis* and generally recognized as safe (GRAS) *E. coli* Nissle as chassis because there is natural biocontainment of the eventual LBTs (i.e., lack of survival). Moreover, the Food and Drug Administration (FDA) has already approved LBTs with these chassis for phase I trials (Braat et al., 2006; Puurunen et al., 2021). Because of the failure of these trials in demonstrating physiological change in human hosts, and that the FDA will not approve LBTs that are ineffective in treating disease, better chassis for functional manipulation of the luminal environment are needed. Moreover, patient advocacy groups such as the Juvenile Diabetes Research Foundation, the National Phenylketonuria Alliance, and the Crohn’s and Colitis Foundation, as well as economists who are concerned about rising healthcare costs (Holman, 2020; Martin et al., 2021), are encouraging scientists to investigate therapeutics with curative intent. In their guidance to industry, the FDA stated that LBTs “(M)ay be isolated from human hosts… (with the goal) to stimulate other potentially beneficial cellular processes as a result of transient persistence and/or long-term colonization with the microorganisms” (Food and Drug Administration, 2016). Hence, engineered native bacteria fit the FDA’s definition of LBTs. Recent therapies with CAR-T already demonstrate the FDA’s willingness to approve therapies where cells from individuals are modified and used, autologously, to treat chronic or debilitating diseases (June and Sadelain, 2018). Hence, the FDA already has language in their guidance regarding LBTs, as well as precedence in autologous cell-based therapeutics that support the potential use of engineered native bacteria for the treatment of chronic diseases. Nevertheless, biocontainment will be an important issue for engrafting LBTs such as engineered native bacteria. Though native chassis demonstrate natural biocontainment (as demonstrated in Figures 2C and 2D) additional kill-switch biological circuits (Stirling et al., 2017) may need to be employed prior to use in humans. Even if engineered native bacteria are not a clinically viable path, they have inherent value as a tool for understanding the microbe-host relationship and for performing more mechanistic studies to better understand how specific bacterial functions affect host physiological processes.

The central novelty of engineered native *E. coli* is that they demonstrate: (1) stable, long-term engraftment of low-abundance organisms in the absence of antibiotic pre-treatment or selection; (2) persistent metabolomic change from a single treatment; (3) persistent physiological change from a single treatment; and (4) reversal of the pathological phenotype after a single treatment. The strategy of using native bacteria as a chassis to introduce transgenes of interest can be used for different organ systems (e.g., skin, lungs, vagina), expanding our ability to perform mechanistic studies and providing a strategy for the creation of therapeutic engineered native bacteria in any microbiome- mediated/modulated disease. Moreover, personalized engineered native bacteria will help overcome the barriers that have limited the use of engineered bacteria in humans, including interpersonal differences and micro-niche changes induced by disease. Finally, life-long compliance with chronic therapies is a substantial burden in patients with chronic diseases, which contributes to their poor morbidity and mortality—and to rising healthcare costs. Some engineered probiotics in development for disease intervention require multiple doses (sometimes up to 3 times daily), which may add significant complications for feasibility, cost, and adherence. If the engineered native bacteria strategy is successfully developed and translated to humans, it has the potential to introduce novel, curative biotherapeutics that improve treatment of chronic diseases without relying on patient compliance.

### Limitations of the study

Our analysis of isolated human native *E. coli* demonstrates that these bacteria are quite resistant to genetic manipulation. However, several recent publications report more effective methods of engineering native bacteria (Brophy et al., 2018; Jin et al., 2022; Rubin et al., 2022; Wiles et al., 2018). Any function engineered into native bacteria will likely reduce the fitness of bacteria unless there is selective pressure to maintain the transgene of interest. Moreover, some genes may be more burdensome than others. Nevertheless, in these proof-of-concept studies we have demonstrated that, even without selective pressure, native *E. coli* have the potential to maintain an engineered function for years. IL-10 and BSH production are likely not burdensome enough to affect chassis fitness and native *E. coli* are resilient enough to continue to constitutively express the transgenes of interest. However, whether this observation can be generalized to all other functions potentially introduced with native *E. coli* chassis is unlikely. Moreover, it is unclear whether other native chassis are as resilient, and thus would elicit the same physiological response, as the native *E. coli* described in this study.

## STAR★METHODS

Detailed methods are provided in the online version of this paper and include the following:

### RESOURCE AVAILABILITY

#### Lead contact

Further information and requests for resources should be directed to and will be fulfilled by the lead contact, Amir Zarrinpar (azarrinpar@ucsd.edu).

#### Materials availability

EcAZ-1, EcAZ-2, EcAZ-2^BSH+^, and EcAZ-2^IL10+^ will be made available subject to a materials transfer agreement with the University of California, San Diego.

#### Data and code availability

Data have been deposited at the European Nucleotide Archive under accession number ENA:PRJEB39003 and are publicly available as of the date of publication. Proteome data was uploaded to massive.ucsd.edu and can be accessed using the following identifiers: PXD032187.

All original code has been deposited at https://github.com/ZarrinparLab/2022_enb_paper and is publicly available as of the date of publication.

Any additional information required to reanalyze the data reported in this paper is available from the lead contact upon request.

### EXPERIMENTAL MODEL AND SUBJECT DETAILS

#### Animal Experiments

All animal experiments were conducted in accordance with the guidelines of the IACUC of the University of California, San Diego. C57BL/6 mice (Jackson Laboratories) or B6.Cg-*Lep*^*ob*^/J (Jackson Laboratories) were housed in a specific pathogen free facility (irradiated chow and autoclaved bedding), or when specified, in a separate section of the vivarium that was a low barrier facility (non-irradiated chow and non-autoclaved bedding; e.g., Figures 1A and 1D). Mice were housed 4–5 per cage unless otherwise stated. They were fed a normal-chow diet (Diet 7912, Teklad Diets, Madion, WI). After an acclimation period, 12-week-old male mice were pseudorandomized into two groups. This pseudorandomization was based on the initial weight of the mice; in the end, the mean and standard deviation of the weight of the mice was the same between all groups. Mice were gavaged with 200 μL of either PBS or ~1×10^10^ cfu/mL of the corresponding bacterial strain unless otherwise stated. Body weight and food consumption were monitored every 1–2 weeks. In the experiment including mice on a 0% fat diet, weight was monitored more frequently, and the study was terminated due to poor health (e.g. low weight, instances of bone fractures) of the animals in accordance with our Animal Care Protocol.

#### Human volunteers

For human-derived strains, volunteers undergoing outpatient endoscopy were selected in accordance with VA San Diego IRB #H130266. Meta data including sex, gender identity, and age of the participants were not recorded as they did not pertain to the goal of the study.

### METHOD DETAILS

#### Bacterial Isolation

For murine derived strains; stool was collected from a male C57BL/6 male mouse acquired from Jackson Laboratory (Bar Harbor, ME). Stool was suspended in sterile DI water and homogenized for 2 min. at 3500 rpm in Mini-Beadbeater-24 (Biospec, Bartlesville, OK). Homogenized stool was plated on MacConkey agar containing lactose. Potential *E. coli* colonies were selected based on colony shape and ability to ferment lactose. They were cultured for isolation twice. 16S rRNA genes were amplified using the primers described below (Illumina Part #15044223 Rev. B) and sequenced using Sanger sequencing (Eton Biosciences, La Jolla, CA) to confirm identity.

For human-derived strains, volunteers undergoing outpatient endoscopy were selected in accordance with VA San Diego IRB #H130266. Biopsies were collected from various regions of the GI tract adjacent to sites that were needed for clinical indications (e.g. polyps) and immediately transferred to sterile PBS. Tissue samples were homogenized, and bacterial growth, isolation, and identification was performed as described above. 16S rRNA gene amplicon PCR forward primer =

5′-TCGTCGGCAGCGTCAGATGTGTATAAGAGACAGCCTACGGGNGGCWGCAG;

16S amplicon PCR reverse primer =

5′-GTCTCGTGGGCTCGGAGATGTGTATAAGAGACAGGACTACHVGGGTATCTAATCC

(Klindworth et al., 2013).

#### Strain Development

The index native murine *E. coli* used for transformation was labeled as EcAZ-1. Genome assembly for this strain can be found at the European Nucleotide Archive under accession number ENA:PRJEB39003. Pacbio and Illumina reads were assembled using Unicycler (https://doi.org/10.1371/journal.pcbi.1005595) v.0.5.0 using default settings and annotated with Prokka (https://doi.org/10.1093/bioinformatics/btu153) v.1.14.6 with an assigned species of *Escherichia coli* and adding Swissprot proteins (downloaded 6/16/2021) as an additional annotation source. For EcAZ-2, ECAZ-1 was exposed to P1*vir* lysate trained on MG1655 with kanamycin resistance (*aph*) and fluorescence (*GFP-mut2*) cassette added at the attB site using recombination (pSIM18). Successful transductants were selected for using kanamycin resistance (*aph*) and fluorescence (EcAZ-2). EcAZ-1 was nearly 100-fold more resistant to phage transduction than *E. coli* Nissle. For the construction of EcAZ-2 containing bile salt hydrolase (BSH; EcAZ-2^BSH+^), EcAZ-1 was exposed to P1*vir* lysate trained on MG1655 containing chloramphenicol resistance (*cat*) and *bsh* cassette added at the yfgG site using recombination (pSIM18). Successful transductant was selected for functionality of BSH and chloramphenicol resistance. Verification of BSH functionality was verified by plating on lactose-containing LB plates supplemented with TDCA. Precipitation of DCA indicated functioning BSH. The successful transductant was then exposed to lysate with kanamycin resistance (*aph*) and fluorescence (*GFP-mut2*). Successful transductant was selected for gain of kanamycin resistance. The *bsh* gene used for this study naturally occurs in *Lactobacillus salivarius* JCM1046 and is effective at altering bile acids in germ free and antibiotic-treated, conventionalized mice when expressed by an *E. coli* Nissle chassis (https://doi.org/10.1073/pnas.1323599111). The first insertion event (for the cassette containing *aph* and *gfp*) occurred between chromosomal bases 3,267,361–3,274,418, which covers a Ribosomal RNA operon, *murI* (glutamate racemase) and *btuB* (vitamin B12 / E colicin / bacteriophage BF23 outer membrane porin BtuB). The second insertion event (for the cassette containing *cat* and *bsh*) occurred between chromosomal bases 1,886,038–1,887,917, which covers *ydcM* (putative transposase), *ybhB* (putative kinase inhibitor), and *ybhC* (outer membrane lipoprotein).

For the construction of EcAZ-2 containing IL-10 (EcAZ-2^IL10+^), EcAZ-1 was exposed to a lysate trained on MG1655 containing chloramphenicol resistance (*cat*) and human *Il10*. The successful transductants were selected for via IL-10 gene detection by PCR and chloramphenicol resistance. Insertion occurred in the *yfgG* (metal ion stress response protein) gene. Verification of IL-10 expression was performed by growing a overnight cultures of 6 isolates per mouse in Super Optimal Broth (SOB), 12.5 ug/mL of Kanamycin and 10 ug/mL of chloramphenicol. The OD600 of the cultures were read and the lowest value was used to normalize the amount of bacteria used on the ELISA. The normalized cultures were centrifuged and and resuspended in Assay diluent (10% FBS in 1× PBS). The samples underwent four freeze-thaw cycles and the cell lysates were tested on a human IL-10 detection ELISA (Catalog 430601, Biolegend). The supernatant that was drawn off each sample before normalization was also tested on a human IL-10 detection ELISA (Catalog 430601, Biolegend).

#### Growth Curves

Overnight cultures of SOB, supplemented with no antibiotic for EcAZ-1, 12.5 ug/mL of kanamycin for EcAZ-2, and 10 ug/mL of chloramphenicol for EcAZ-2^BSH+^ and EcAZ-2^IL10+^, were back diluted 1:100 in the same media described and loaded in triplicate in a 96-well plate. Blanks for each media were also loaded in triplicate. Incubation and measurements were done in an Infinite M Nano+ plate reader (Teacn, Morrisville, NC) at 37°C. OD600 measurements were performed every 10 minutes for 18 hours directly after 5 seconds of shaking. OD was determined by subtracting out the average blank for the media type for each time point.

#### Antibiotic Susceptibility Testing

Human bacterial isolates were assessed for susceptibility to commonly used laboratory antibiotics chloramphenicol, kanamycin, and carbenicillin. Overnight cultures of each strain were grown in LB broth. Antibiotics were diluted in Mueller Hinton broth to the following working concentrations: (1) 10 ug/mL, 5 ug/ml, and 2.5 ug/mL of chloramphenicol, (2) 25 ug/mL, 12.5 ug/mL, and 6.25 ug/mL of kanamycin, (3) 100 ug/mL, 50 ug/mL, and 25 ug/mL. Overnight cultures were back diluted 1:1000 into Mueller Hinton broth with antibiotics in a 96-well plate and incubated at 37°C for 18 hours. OD600 measurements were performed at the beginning and end of the 18 hour incubation directly after 5 seconds of shaking. OD was determined by subtracting out the average blank for the media type for each time point.

#### Transformation Efficiency

Human strains were tested for susceptibility to transformation via electroporation. Electrocompetent cells were prepared in 1mM MOPS buffer. Three plasmid sizes were assessed included pTrcHisC (4.4kb), a modified version of pNCM (pNCM[ΔccdB::aadA Δrep::[cat gfp]], roughly 70kb), and an additionally modified version of pNCM (pNCM[traMJ::[aph FRT] Δtra ΔfinO202::[N25-gfp]], roughly 35kb). All plasmid transformations were assessed at 1800 V, 200 ohms, and 25 uF. Strains were recovered in SOC for 1.5 hours and plated on selective and non-selective LB plates. After overnight incubation at 37°C, colonies were counted and CFU/mL was determined for successful transformants and viable cells present. Efficiency was determined as CFU/mL on selective plates divided by CFU/mL on non-selective plates. Due to complications with antibiotic selection for the modified version of pNCM, some transformation efficiencies could not be calculated. In these instances, the limit of detection is reported.

#### Conjugation Efficiency

Human bacterial isolates were assessed for susceptibility to conjugation using a 2,6-diaminopimelic acid dependent donor strain containing a modified F-plasmid, pNCM, containing a kanamycin resistance marker and green fluorescent protein with a deletion in *finO* to promote conjugation. The human and donor strains were grown to mid-log phase growth (OD600 0.4–0.6) in SOC. The human strains were each mixed 1:1 with the donor strain and incubated in SOC with no shaking for 4 hours. Successful conjugates were selected on LB plates for the ability to grow without DAP, and for a gain of GFP and kanamycin resistance. After overnight incubation at 37°C, colonies were counted and CFU/mL was determined for successful conjugants and viable cells present. Efficiency was determined as CFU/mL on selective plates divided by CFU/mL on non-selective plates.

#### Colonization Assessment

For stool, we collected 3–5 stool pellets from individual mice and weighed them. 1 mL of sterile deionized water was added to each sample along with one sterile chrome bead (Neta Scientific, Hainesport, NJ). Samples were homogenized for 2 min. at 3500 rpm in Mini-Beadbeater-24 (Biospec, Bartlesville, OK). For control samples, homogenized stool was plated on kanamycin and chloramphenicol to check for bacterial contamination. For EcAZ-2 colonized mice, samples were plated on chloramphenicol to check for unintended bacterial contamination and diluted and plated on kanamycin to calculate CFU/gram of stool. The EcAZ-2^BSH+^ and EcAZ-2^IL10+^ stool was diluted and plated on kanamycin and chloramphenicol.

Collected and tested tissues include duodenum defined as the first 10 cm of small intestine, jejunum defined as the next 10 cm of small intestine, Ileum defined as the next 5 cm of small intestine, cecum, and colon.

For tissues, mice were euthanized and tissue samples were harvested and immediately placed on ice. Dissection tools were disinfected between animals. Samples were processed and plated as described for stool. For each region of the GI tract, bacterial colonies were tested for enzyme functionality by patching from non-selective plates onto lactose and taurodeoxycholic acid (TDCA) containing plates. Up to 25 colonies for each region for each mouse were tested. In cases where 25 colonies did not grow, all colonies were tested.

For each region of the GI tract, verification of IL-10 expression was performed by growing an overnight culture of isolates in SOB, 12.5 ug/mL of Kanamycin and 10 ug/mL of chloramphenicol. In order to lyse the bacteria, the cultures were centrifuged, and the resulting pellet was resuspended in Assay diluent (10% FBS in 1× PBS). The samples were then flash frozen and defrosted for 4 rounds to lyse the cells and used on a IL-10 detection ELISA (Catalog 430601, Biolegend).

#### Co-Housing Experiment

Three pairs of 6-month-old male C57BL/6 mice were co-housed in a low barrier facility (where food and bedding were not autoclaved). From each cage, one mouse was gavaged with 200 μL of PBS while the other was gavaged with 200 μL ~1×1010 cfu/mL EcAZ-2. After gavage, mice were single housed for a week. Five days post-gavage, baseline stool was collected from each mouse. The pairs were then reunited with their cage mate. Initial stool collection demonstrated coprophagia but no persistent colonization. After 19 days, the protocol was modified so that mice were separated and individually housed one day prior to stool collection. After stool collection, each mouse was reunited with their cage mates until the next stool collection. Thus, with the revised protocol, coprophagic incidents would no longer appear and only incidents of horizontal transfer/colonization would be detectable.

#### Dosing experiment

20 mice were singly housed with 5 mice per condition. All mice were house in the low barrier facility. Mice were gavaged with 200 μL of either ~1×10^8^ cfu/mL, 1×10^6^ cfu/mL, 1×10^4^ cfu/mL, or 1×10^2^ cfu/mL of EcAZ-1. Stool collections were performed 2 days, 5 days, 1 week, 2 week, and 20 days after gavage as described above.

#### Gnotobiotic Mice

Germ-free C57BL/6 mice were bred and maintained in sterile flexible film isolators and screened for bacterial, viral, and fungal contamination as described (https://doi.org/10.30802/AALAS-JAALAS-18-000130). Mice were fed autoclaved chow (Envigo) and transferred into the Sentry SPP cage (Allentown). Mice were inoculated with corresponding bacteria oral gavage. Stool was collected for bile acid analysis 7 days later. Mono-colonization was confirmed upon euthanasia of the gnotobiotic mice.

#### Quantitative Multiplex Proteomics

For sample preparation, cell pellets from vehicle, EcAZ-2, and EcAZ-2^BSH+^ *E. coli* were analyzed by quantitative multiplex proteomics at the UCSD Collaborative Center for Multiplexed Proteomics (https://doi.org/10.1016/j.cell.2020.07.040). EcAZ-1, EcAZ-2, and EcAZ-2^BSH+^ were grown overnight in LB with their respective antibiotics in replicate then back diluted 1:20 into 10mL LB broth and grown to mid-log phase (OD600 0.4–0.6). Samples were resuspended in equal volumes of lysis buffer (6M urea, 7% SDS, 50 mM TEAB, titrated to pH 8.1 with phosphoric acid) and sonicated. Disulfide bonds were reduced in 5 mM dithiothreitol (DTT) at 56 °C for 30 min and free cysteines were alkylated in 15 mM iodoacetamide (IAA) in a darkened environment for 20 min. The alkylation reaction was quenched for 15 min at room temperature through the addition of an equivalent volume of DTT as in the reduction reaction. Proteins were then trapped using ProtiFi S-Trap columns, digested with trypsin (Promega, V5113), and eluted according to manufacturing protocols. Eluents were desalted on C18 columns using instructions provided by the manufacturer (Waters). Desalted samples were dried under vacuum. Samples were subjected to peptide quantification using a Pierce Colorimetric Peptide Quantification Assay kit per the manufacturer’s instructions. 50 μg of each sample was separated for further processing, with four samples aliquoted twice to reach a total of 16 samples.

For sample labeling, samples were resuspended in a solution of 30% anhydrous acetonitrile (ACN) with 200 mM HEPES, pH = 8.5. Tandem mass tag (TMT) labels (Thermo Fisher Scientific; Catalog Number: A44520, Lot Number: WJ327115) were suspended in anhydrous ACN to a final concentration of 20 mg/mL, and 7 μL were added to each resuspended sample. Samples were randomized in the TMT 16plex. The labeling reaction was allowed to proceed for 1 h at room temperature, after which excess label was quenched through the addition of 8 μL of 5% hydroxylamine for 15 min. 50 μL of 1% trifluoroacetic acid (TFA) was added to each sample, and samples were combined and desalted on C18 columns (Waters) as above. The multiplexed samples were dried under vacuum.

For Fractionation, multiplexed samples were next subjected to fractionation using reverse phase high pH liquid chromatography. Briefly, samples were fractionated on an Ultimate 3000 high performance liquid chromatography system fitted with fraction collector, C18 column (4.6 × 250 mm), solvent degasser, and variable wavelength detector. Multiplexed samples were fractionated on a gradient ranging from 22% to 35% ACN with 10 mM ammonium bicarbonate (ABC) over 60 min. The resulting 96 fractions were concatenated using methods previously described (https://doi.org/10.1002/pmic.201000722). Briefly, alternating wells were combined within each column, resulting in 24 total fractions. Alternating concatenated fractions were used for proteomic analysis.

For mass spectrometry-based proteomic analysis, all proteome mass spectrometry data were collected on an Orbitrap Fusion mass spectrometer (Thermo Fisher Scientific) with an in line Easy-nLC. Previously described methods were utilized for data collection (https://doi.org/10.1038/s41380-021-01339-z). Briefly, proteome data was collected using a “low resolution” method.

For data processing and normalization, raw files were searched using the SEQUEST algorithm in Proteome Discoverer 2.5 against the reference proteome for *E. coli* strain NGF-1 with the BSH1 sequence added (Uniprot Accession Q1WR93). Data were searched using a precursor mass tolerance of 50 ppm and fragment mass tolerance of 0.6. Static modifications were specified as follows: TMTpro on lysines and N-termini and carbamidomethylation of cysteines. Dynamic modifications were specified to include oxidation of methionines. Resulting peptide spectral matches were filtered at a 0.01 FDR by the Percolator module against the decoy database. Peptide spectral matches were exported, summed to the protein level, and batch corrected as previously described (https://doi.org/10.1074/mcp.M116.066019). Following batch correction, each sample normalized using Box-Cox power transformation (MASS R package, version 7.3–55), abundances were scaled between 0–1 (reshape R package, version 0.8.8), and each sample was standardized by its mean (https://doi.org/10.1111/j.2517-6161.1964.tb00553.x). Sample duplicates were averaged at this step. Final values represent normalized relative protein abundances.

#### BSH Activity Assay

Overnight cultures of LB, supplemented with no antibiotic for EcAZ-1, 12.5 ug/mL of kanamycin for EcAZ-2, and 10 ug/mL of chloramphenicol for EcAZ-2^BSH+^ were back diluted 1:20 in 1mL LB with the appropriate antibiotics and grown to mid-log phase (OD600 0.4–0.6). Cultures were centrifuged and the cell pellet was resuspended in 1mL of 0.1M sodium phosphate buffer, pH 6.0. Bacteria underwent freeze-thaw lysis and spun to pellet cell debris. Supernatant was mixed with 50uM TDCA and aliquots were removed and quenched in equal volume of 0.5M HCl at each time point. Samples were stored at −80°C until mass spectroscopy analysis.

#### Stool and Serum Bile Acids

Bile acids were extracted from samples as described before (https://doi.org/10.1038/s41467-018-05336-9, https://doi.org/10.1007/s00216-016-0048-1). Briefly, stool samples were homogenized and extracted in methanol (10 mg of sample/100 μL) containing heavy internal standards. Serum samples (25 μL) were extracted with 75 μL of methanol containing heavy internal standards. After vortexing for 10 minutes and centrifuging (16,000 × g, 4 C, 10 min), supernatants were transferred to glass vials for injection. Bile acids were analyzed on a Dionex Ultimate 3000 LC system (Thermo) coupled to a TSQ Quantiva mass spectrometer (Thermo) fitted with a Kinetex C18 reversed phase column (2.6 μm, 150 × 2.1 mm i.d., Phenomenex). The following LC solvents were used: solution A, 0.1 % formic acid and 20 mM ammonium acetate in water, solution B, acetonitrile/methanol (3/1, v/v) containing 0.1 % formic acid and 20 mM ammonium acetate. The following reversed phase gradient was utilized: at a flow rate of 0.2 mL/min with a gradient consisting of 25–29 % B in 1 min, 29–33 % B in 14 min, 33–70 % B in 15 min, up to 100 % B in 1 min, 100 % B for 9 min and re-equilibrated to 25 % B for 10 min, for a total run time of 50 min. The injection volume for all samples was 10 μL, the column oven temperature was set to 50°C and the autosampler kept at 4°C. MS analyses were performed using electrospray ionization in positive and negative ion modes, with spray voltages of 3.5 and −3 kV, respectively, ion transfer tube temperature of 325°C, and vaporizer temperature of 275°C. Multiple reaction monitoring (MRM) was performed by using mass transitions between specific parent ions into corresponding fragment ions for each analyte. Results were quantified using isotopically labeled internal standards. Data were averaged across samples.

The bile acids measured using this technique include CA, TCA, bMCA, TbMCA, α-muricholic acid (aMCA), tauro-α-muricholic acid (TaMCA), chenodeoxycholic acid (CDCA), taurochenodeoxycholic acid (TCDCA), DCA, TDCA, hyocholic acid (HCA), taurohyocholic acid (THCA), ω-muricholic acid (oMCA), tauro-ω-muricholic acid (ToMCA), ursodeoxycholic acid (UDCA) and lithocholic acid (LCA). Glycine conjugated bile acids were not detected. Of note, no UDCA was detected in the stool. Total bile acid measurements was the sum of all bile acids measured using mass spectrometry. Primary bile acids included CA, TCA, aMCA, TaMCA, bMCA, TbMCA, CDCA, and TCDCA. Secondary bile acids included DCA, TDCA, oMCA, ToMCA, UDCA, and LCA.

#### 16S rRNA Gene Sequence/Microbiome Data Processing and Analysis

16S rRNA gene amplicon sequences were processed using Qiime2 (version 2020.6) using default parameters (https://doi.org/10.1038/s41587-019-0209-9). ASVs were generated by the Deblur method, trimmed to 120bp (https://doi.org/10.1128/mSystems.00191-16). Taxonomy was assigned via the sklearn plugin (https://doi.org/10.1101/2020.10.05.326504; https://doi.org/10.1186/s40168-018-0470-z) against the Silva v123 model (https://doi.org/10.1093/nar/gks1219), and ASVs were filtered to remove Eukaryotic, Mitochondrial, Plastid, and unassigned taxa. The phylogenetic diversity metrics and PCoA were then calculated on samples rarefied to 4000 reads.

#### qPCR from Liver and Terminal Ileum

The livers and terminal ileum from control and EcAZ-2^BSH+^ mice were flash frozen in liquid nitrogen. The frozen tissues were homogenized and RNA was extracted using TriZol reagent according to manufacturer’s instructions. 1ug of RNA was reverse transcribed using quanta biosciences qScript cDNA supermix (Cat# 95048–025). The samples were diluted 1:50 and qRTPCR was run using FastStart Universal SYBR Green Master (Rox) (Cat# 4913850001) on an Applied Biosystems QuantStudio 5. The data were analyzed and gene expression was expressed relative to mrpl46.

**Table:** 

Primers	Sequence

CYP7a1 Fwd	GGGATTGCTGTGGTAGTGAGC
CYP7a1 Rev	GGTATGGAATCAACCCGTTGTC
CYP27a1 Fwd	CCAGGCACAGGAGAGTACG
CYP27a1 Rev	GGGCAAGTGCAGCACATAG
Fxr Fwd	GCTTGATGTGCTACAAAAGCTG
Fxr Rev	CGTGGTGATGGTTGAATGTCC
Nr0b2 Fwd	TGGGTCCCAAGGAGTATGC
Nr0b2 Rev	GCTCCAAGACTTCACACAGTG

#### Blood Collection and Serum Biomarker Measurements

Blood was collected from fasted (16 h) or re-fed animals (30 min). Re-feeding was performed by oral gavage of Ensure (Abbott, Columbus, Ohio). In male mice, serum insulin was performed using the Bio-Plex Pro Mouse Diabetes 8-Plex Assay (Bio-Rad, Hercules, CA) as per the manufacturer’s protocol.

#### Glucose Tolerance

Glucose tolerance assays were performed on fasted mice (16 h, Pur-O-Cel bedding) by monitoring glucose levels after a glucose bolus (10 uL of 20% glucose/kg of body weight (BW)) via oral gavage. Glucose concentration was measured from a tail snip using a OneTouch Ultra glucometer. Tail tips were anesthetized with a topical anesthetic (Actavis, Parsippany-Troy Hills, NJ) prior to snip.

#### Insulin Measurement

10-week old female mice were fasted (5 h, Pur-O-Cel bedding) and then bled from the tail vein to collect fasted plasma. Two days later, mice were again fasted (14–16 h, Pur-O-Cel bedding) and then orally gavaged with 0.5g glucose/kg body weight of Ensure (Abbott, Columbus, Ohio) to measure insulin response to a mixed meal. 30 minutes after the gavage, blood was collected through a submandibular bleed. Serum insulin from fasted and fed blood was measured using a mouse ELISA (Crystal Chem Ultra Sensitive Mouse ELISA Kit).

### QUANTIFICATION AND STATISTICAL ANALYSIS

All comparisons of EcAZ-2 to EcAZ-2^BSH+^ were calculated using Mann-Whitney U test or Kruskal-Wallis test with post-hoc Dunn’s multiple comparison test as specified with the exception of Figures 1E, 1F, 4D, and S3A–S3C, which used a student’s t-test due to low sample size. When student’s t-test was used, normality of data was confirmed with a Q-Q plot prior to the test. For qualitative 2×2 comparisons (Figures 2B and S2B), a Fisher’s exact test was used to determine significance.

For proteomics data, statistical tests assuming normalized data with equal means and unequal variances were used. To compare all three groups, Brown-Forsythe and Welch one-way ANOVA was used. A p-value of < 0.05 was considered statistically significant. Protein abundances were graphed using GraphPad Prism software (version 9.3.1).

## Supplementary Material

1

## Figures and Tables

**Table 1.** Human-derived *E. coli* isolated from intestinal biopsies are genetically tractable

Metadata			Antibiotic MIC (mg/mL)			Transformation (% efficiency)			Conjugation
Strain	Host species	Tissue	Cm	Kan	Carb	Small plasmid (4.4 kb)	Medium plasmid (35 kb)	Large plasmid (70 kb)	Efficiency (or limit of detection)

EcAZ-1	mouse (C57BL6)	feces	5	6.25	25	4.25	4.51E–4	6.60E–5	5.98E–3
AZ-61	human-2	rectum	5	6.25	50	0.067	1.97E–4	1.99E–5	1.31E–5
AZ-116	human-4	ileum	10	6.25	resistant	-	2.58E–6	2.42E–5	2.58E–3
AZ-120		cecum	5	6.25	25	0.23	1.18E–6	1.44E–5	5.13E–3

Abbreviations: Cm, chloramphenicol; Kan, kanamycin; Carb, carbenicillin; see also Figure S5.

**KEY RESOURCES TABLE:** 

REAGENT or RESOURCE	SOURCE	IDENTIFIER

Bacterial and virus strains		

*Escherichia coli* MG1655	Coli Genetic Stock Center	Cat#: 6300
*Escherichia coli* Nissle 1917	Provided by Suckjoon Jun	N/A
*Escherichia coli* EcAZ-1	This manuscript	N/A
*Escherichia coli* EcAZ-2	This manuscript	N/A
*Escherichia coli* EcAZ-2^BSH^	This manuscript	N/A
*Escherichia coli* EcAZ-2^IL10^	This manuscript	N/A
*Escherichia coli* AZ-61	This manuscript	N/A
*Escherichia coli* AZ-116	This manuscript	N/A
*Escherichia coli* AZ-120	This manuscript	N/A
*Escherichia coli* AZ-122	This manuscript	N/A
P1 *vir*	Coli Genetic Stock Center	Cat#: 12133

Chemicals, peptides, and recombinant proteins		

Sodium taurodeoxycholate hydrate (TDCA)	Millipore Sigma	Cat#:T0875
2,6-diaminopimelic acid	Life Technologies	Cat#:B22391.03
Tandem mass tag labels	Thermo Fisher Scientific	Cat#: A44520
qScript cDNA supermix	Quantabio	Cat:# 95048-025
FastStart Universal SYBR Green Master	Sigma-Aldrich	Cat#: 4913850001

Critical commercial assays		

IL-10 ELISA	Biolegend	Cat#: 430610
Bio-Plex Pro Mouse Diabetes 8-Plex Assay	Bio-Rad	Cat#: 171F7001M
Ultra Sensitive Mouse ELISA Kit	Crystal Chem	Cat#: 90080

Deposited data		

Proteomics data	This manuscript: massive.ucsd.edu	PXD032187
16S sequences and genome assembly	ENA project	PRJEB39003

Experimental models: Organisms/strains		

C57BL/6J mice	The Jackson Laboratory	Strain #000664
*Ob/Ob* mice B6.Cg-*Lep*^*ob*^/J	The Jackson Laboratory	Strain #000632

Oligonucleotides		

16S rRNA gene amplicon PCR forward primer: 5’TCG TCGGCAGCGTCAGATGT GTATAAGAGACAGCCTAC GGGNGGCWGCAG	Klindworth et al., 2013	https://doi.org/10.1093/nar/gks808
16S amplicon PCR reverse primer: 5’GTCTCGTGGGC TCGGAGATGTGTATAAGAGACAGGACTACHVGGGT ATCTAATCC	Klindworth et al., 2013	https://doi.org/10.1093/nar/gks808
CYP7a1 Fwd: GGGATTGCTGTGGTAGTGAGC	This manuscript	N/A
CYP7a1 Rev: GGTATGGAATCAACCCGTTGTC	This manuscript	N/A
CYP27a1 Fwd: CCAGGCACAGGAGAGTACG	This manuscript	N/A
CYP27a1 Rev: GGGCAAGTGCAGCACATAG	This manuscript	N/A
Fxr Fwd: GCTTGATGTGCTACAAAAGCTG	This manuscript	N/A
Fxr Rev: CGTGGTGATGGTTGAATGTCC	This manuscript	N/A
Nr0b2 Fwd: TGGGTCCCAAGGAGTATGC	This manuscript	N/A
Nr0b2 Rev: GCTCCAAGACTTCACACAGTG	This manuscript	N/A

Recombinant DNA		

pTrcHisC	Invivogen	Cat#:1324578
pNCM	GeneBank	Acc#: CP011496
Modified pNCM: pNCM[traMJ::[aph FRT] ΔfinO102::[cat N25-gfp]]	This manuscript	N/A

Software and algorithms		

MASS R package version 7.3-55	https://cran.r-project.org/web/packages/MASS/index.html	N/A
reshape R package version 0.8.8	http://had.co.nz/reshape	N/A
Qiime2 version 2020.6	https://doi.org/10.1038/s41587-019-0209-9	N/A
Prism	GraphPad	v9.3.1

Other		

Orbitrap Fusion mass spectrometer	Thermo Fisher Scientific	Cat#:IQLAAEGAAPFADBMBCX
Dionex Ultimate 3000 LC	Thermo Fisher Scientific	LC/MS-5TF-0330
TSQ Quantiva mass spectrometer	Thermo Fisher Scientific	LC/MS-5TF-0330

## Highlights

- Native *E. coli* from the murine and human gut can be engineered for transgene delivery
- Engineered native *E. coli* (EcAZ) engrafts in conventional mice in non-sterile conditions
- Single treatments with EcAZ can be used to induce persistent physiological change
- Single treatments with EcAZ can persistently ameliorate chronic pathological states
