# Supplementary material for: Intestinal transgene delivery with native E. coli chassis allows persistent physiological changes
Source: Cell. Author manuscript; Available in PMC 2022 Sep 12. (PMC9464905; doi:10.1016/j.cell.2022.06.050)
Supplement: 1 [file NIHMS1828086-supplement-1.pdf]

CellPress
OPEN ACCESS

Supplemental figures

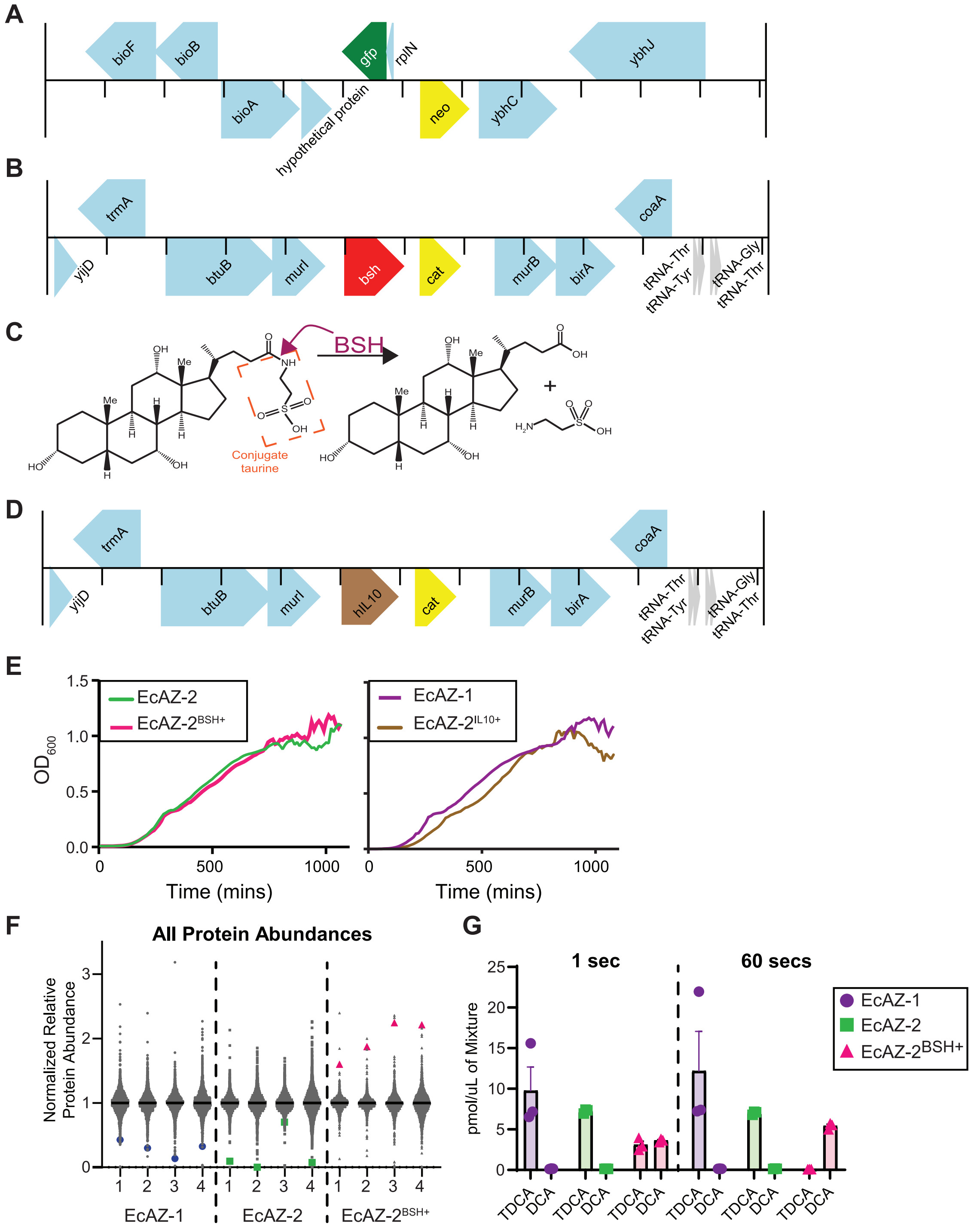

Figure S1. Gut native *E. coli* are genetically tractable and can serve as a chassis for transgene delivery, related to Figure 1

(A) Transgene location of *gfp* in EcAZ-2 as determined by minION sequencing.

(B) Transgene location of *bsh* in EcAZ-2^{BSH+} as determined by minION sequencing.

(legend continued on next page)

(C) Bile salt hydrolase (BSH) is a prokaryotic gene that deconjugates bile acids. Through deconjugation, BSH makes bile acids less polar (and thus unable to be transported by apical sodium-bile acid transporters). The deconjugated bile acids can serve as metabolic substrates for a number of bacteria that then convert them to secondary bile acids.
(D) Transgene location of human IL-10 (hIL10) in EcAZ-2^{IL10+}, as determined by minION sequencing.
(E) Growth curve of EcAZ-2 compared with EcAZ-2^{BSH+} and EcAZ-1 compared with EcAZ-2^{IL10+}. The line represents an average of three measurements per strain.
(F) Proteomic analysis of EcAZ-1, EcAZ-2, and EcAZ-2^{BSH+} for four isolates, demonstrating that although BSH is undetectable in EcAZ-1 and EcAZ-2, it is one of the most highly constitutively expressed proteins in EcAZ-2^{BSH+}.
(G) Raw values used for Figure 1F showing no conversion of TDCA to DCA by EcAZ-1 and EcAZ-2 at 1 and 60 s, but EcAZ-2 converted half of TDCA to DCA at 1 s and all of the TDCA to DCA at 60 s (n = 3).

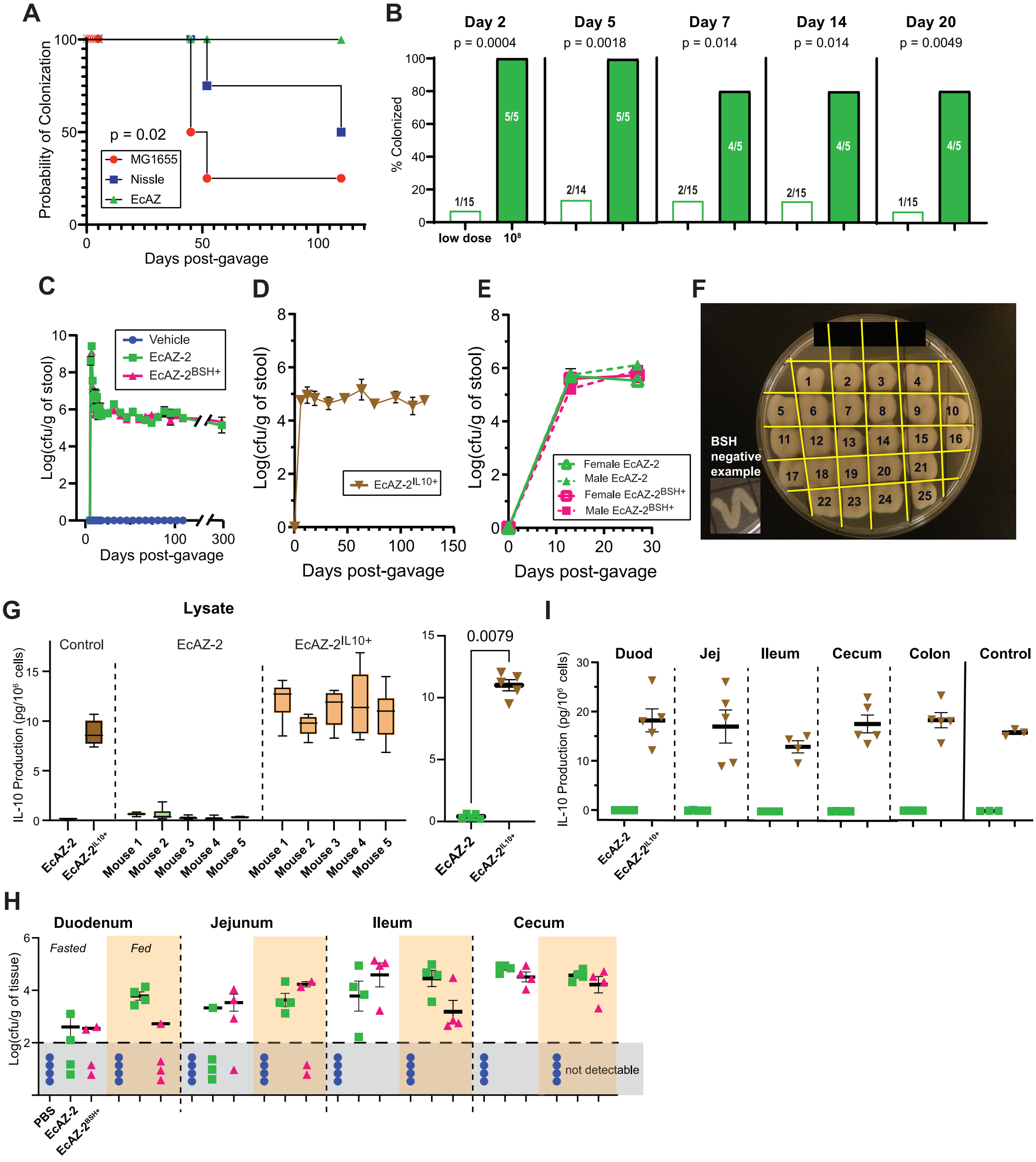

Figure S2. Engineered native *E. coli* can engraft in the luminal environment, related to Figure 2

(A) Percentage of mice colonized with their respective bacteria in a non-sterile, low-barrier mouse facility (4 mice/condition).

(B) Percent of mice colonized with EcAZ-2 after gavage with 10^8 CFUs compared with those that were gavaged with a lower dose. Significance determined with a Fisher's exact test.

(C) Colonization after gavage of 10^{10} CFUs of EcAZ-2 or EcAZ-2^{BSH+} in non-antibiotic-treated CR-WT mice housed in an SPF facility (4–12 mice/condition).

(D) Colonization after gavage of 10^{10} CFUs of EcAZ-2^{IL10+} in non-antibiotic-treated CR-WT mice housed in an SPF facility (5 mice).

(E) Colonization after gavage of 10^{10} CFUs of EcAZ-2 or EcAZ-2^{BSH+} in non-antibiotic-treated CR-WT male and female mice housed in an SPF facility (4 mice/condition).

(F) Example of post-euthanasia testing of 25 colonies from the ileum of a single EcAZ-2^{BSH+} mouse plated on LB containing lactose and TDCA. These strains were isolated 112 days post-gavage from mice who received a single gavage of EcAZ-2^{BSH+}. Precipitate around EcAZ-2^{BSH+} is the deconjugated form of TDCA (DCA) and indicates BSH functionality. Inset shows an example of EcAZ-2 bacteria on the TDCA play not forming a precipitate. All colonies isolated from EcAZ-2^{BSH+} mice for this study formed a precipitate, whereas all colonies isolated from EcAZ-2 mice did not.

(G) Five isolates were taken from EcAZ-2^{IL10+} colonies found in the feces of mice depicted in (D) from the 89 days post-gavage time point. IL-10 from the cell lysate of these isolates are depicted here. EcAZ-2 is the negative control, whereas EcAZ-2^{IL10+} depicts IL-10 measured from a strain that was never gavaged into a mouse (positive control; see Figure 1G).

(legend continued on next page)

(H) Colonization of EcAZ-2 and EcAZ-2^{BSH+} in the gastrointestinal tract of CR-WT mice housed in a SPF facility. Measurements were made from mice euthanized after a 16-h fast or 1 h after refeeding. (4 mice/condition.)
(I) IL-10 levels in the cell lysates of bacteria isolates from mice described in (G).
All error bars indicate standard error of the mean. The marker covers some error bars in (C), (D), and (E).

CellPress
OPEN ACCESS

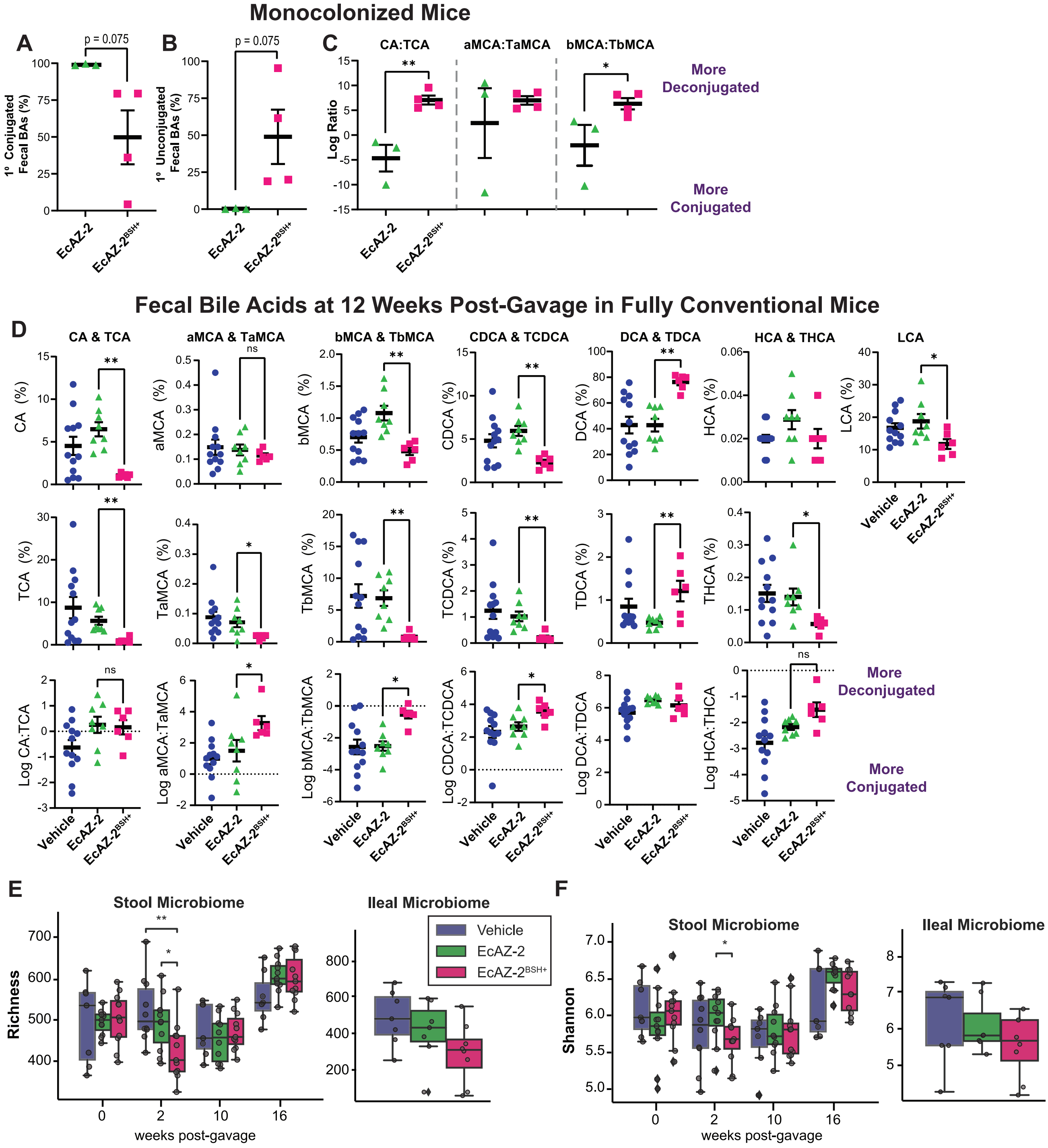

Figure S3. Native *E. coli* can be used to change luminal metabolome without measurable effects in the microbiome, related to Figure 3

(A–C) (A) Primary conjugated fecal bile acids, (B) primary unconjugated fecal bile acids, and (C) log_2 ratio of deconjugated to conjugated bile acids in gnotobiotic mice mono-colonized with either EcAZ-2 or EcAZ-2^{BSH+}. Significant differences determined by Student's t test after normality verified through Q-Q plot.

(D) Deconjugated (top), conjugated (middle), and the log_2 ratio of deconjugated to conjugated fecal bile acids in mice treated with vehicle, EcAZ-2 or EcAZ-2^{BSH+}. Significant differences determined by Kruskal-Wallis test with post hoc Dunn's multiple comparison test comparing EcAZ-2 and EcAZ-2^{BSH+}.

(E and F) (E) Richness and (F) Shannon index from 16S rRNA gene sequencing performed on stool samples collected pre-treatment and at 2, 10, and 16 weeks post-gavage (left) and from the terminal ileum samples at the time of euthanasia (right). Significant differences were determined by Kruskal-Wallis test with post hoc Dunn's multiple comparison test comparing all three conditions.

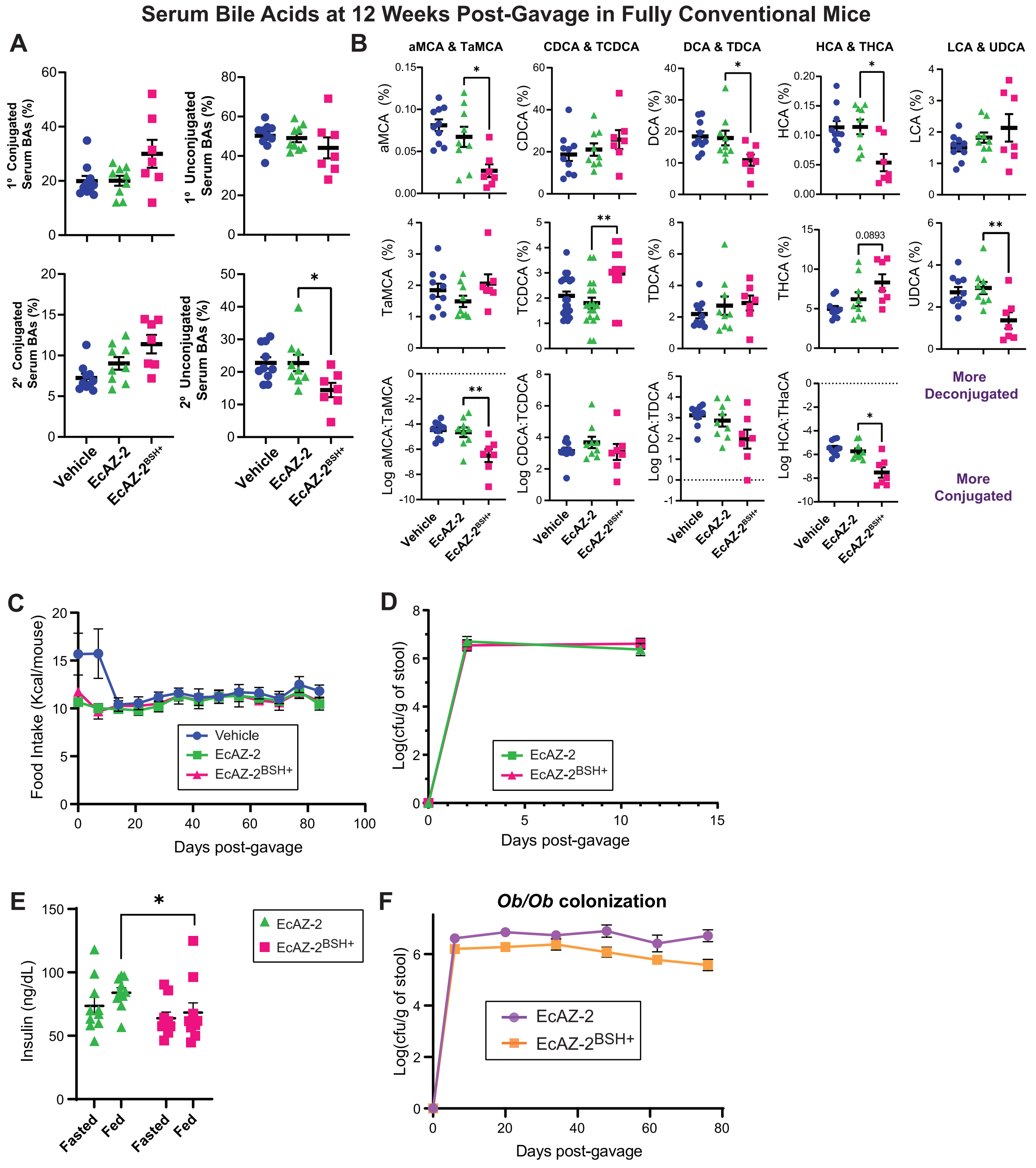

Figure S4. Native *E. coli* can be used to physiologically change the host and treat pathophysiological conditions, related to Figure 4

(A) Primary conjugated, primary unconjugated, secondary conjugated, and secondary unconjugated serum bile acids from mice treated with a single gavage of vehicle, EcAZ-2, or EcAZ-2^{BSH+} 12 weeks prior.

(B) Deconjugated (top), conjugated (middle), and the log_2 ratio of deconjugated to conjugated serum bile acids in mice treated with vehicle, EcAZ-2, or EcAZ-2^{BSH+}.

(C) Food intake in CR-WT mice treated with a single gavage of vehicle, EcAZ-2, or EcAZ-2^{BSH+}.

(legend continued on next page)

CellPress
OPEN ACCESS

(D) Colonization of EcAZ-2 and EcAZ-2^{BSH+} after a single gavage in female CR-WT mice.
(E) Fasting (6 h) and fed (30 min) insulin levels in female CR-WT mice treated with a single gavage of EcAZ-2 and EcAZ-2^{BSH+} 22 days prior.
(F) Colonization of EcAZ-2 and EcAZ-2^{BSH+} after a single gavage in *Ob/Ob* mice.
For (A) and (B), significant differences were determined by Kruskal-Wallis test with post hoc Dunn's multiple comparison test comparing EcAZ-2 and EcAZ-2^{BSH+}.

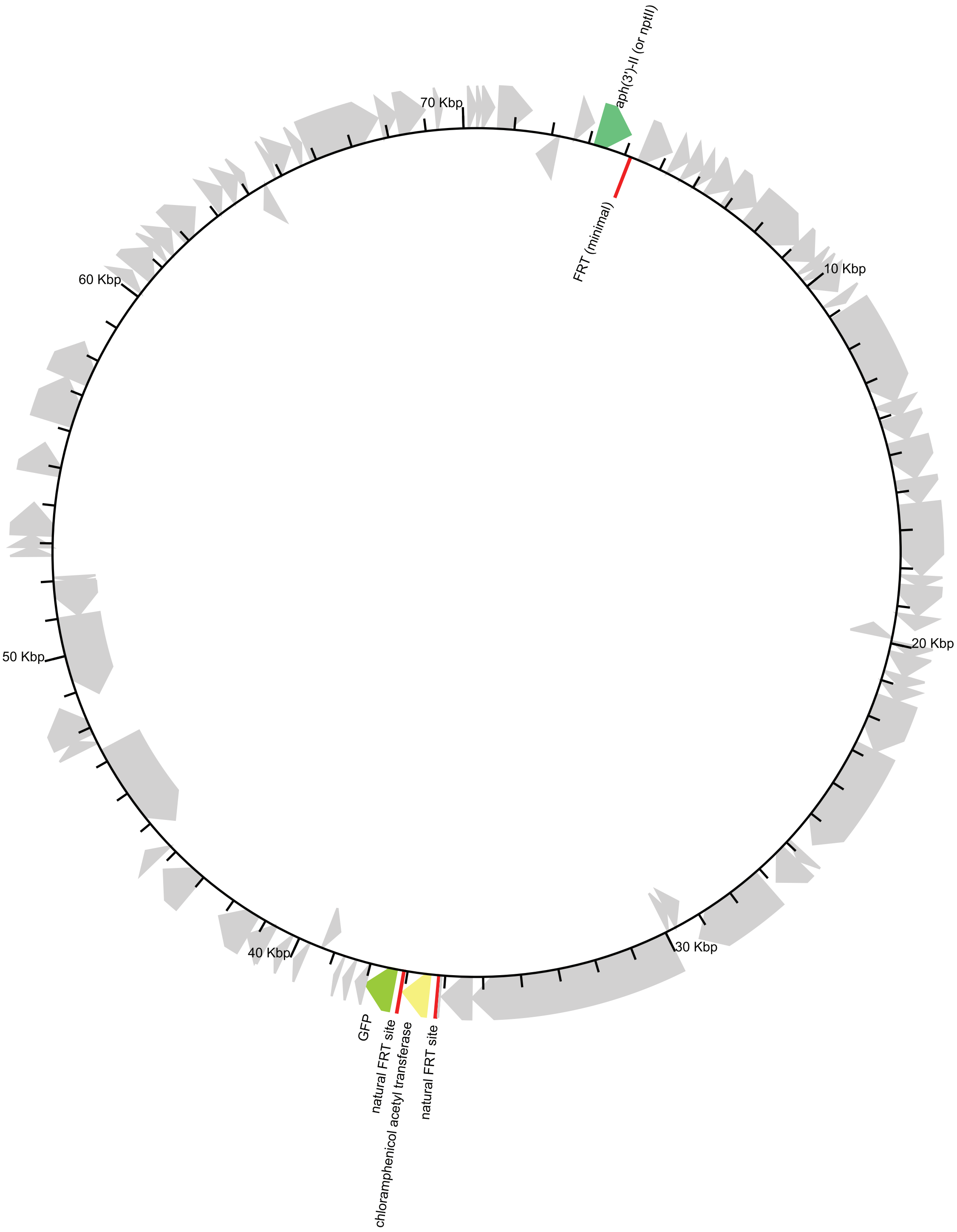

Figure S5. Modified plasmid used for testing tractability in native human *E. coli*; related to Table 1

Modified F-plasmid used for conjugation experiments with native human *E. coli*.
